# ﻿Replacement names for junior homonyms in ants (Hymenoptera, Formicidae)

**DOI:** 10.3897/zookeys.1256.162607

**Published:** 2025-10-16

**Authors:** Brian L. Fisher

**Affiliations:** 1 Department of Entomology, California Academy of Sciences, San Francisco, California, USA California Academy of Sciences San Francisco United States of America

**Keywords:** AntCat, Formicidae, homonymy, ICZN, nomenclature, nomen novum, replacement names, taxonomy

## Abstract

Replacement names are proposed for 40 unresolved junior species group homonyms in Formicidae (Hymenoptera) as required by ICZN Articles 23 and 60. This effort aims to enhance taxonomic stability and facilitate digital management for ant names. In each case, the junior homonym is considered acceptable and in use. To avoid the creation of unnecessary replacement names, names were not proposed for unresolved junior homonyms in cases where 1) the status of the junior homonym is a junior or senior synonym or unidentifiable; 2) the primary homonym pair have not been regarded as congeneric since 1899; or 3) the congeneric placement of the junior secondary homonym is unreliable. The following new names are introduced to replace existing homonyms: *Acromyrmex aurina***nom. nov.**, *Camponotus chucki***nom. nov.**, *Camponotus dorex***nom. nov.**, *Camponotus durnyx***nom. nov.**, *Camponotites flarex***nom. nov.**, *Camponotus robecchii clyne***nom. nov.**, *Camponotus substitutus cylrix***nom. nov.**, *Colobopsis breva***nom. nov.**, *Crematogaster scutellaris gynia***nom. nov.**, *Dolichoderus thoracicus jexina***nom. nov.**, *Ectomomyrmex pylixy***nom. nov.**, *Ectomomyrmex pylor***nom. nov.**, *Euponera tynara***nom. nov.**, *Formica lavateri liora***nom. nov.**, *Formica mytara***nom. nov.**, *Hypoponera rylin***nom. nov.**, *Hypoponera veltan***nom. nov.**, *Lepisiota vynia***nom. nov.**, *Temnothorax flavispinus novira***nom. nov.**, *Messor minor nubina***nom. nov.**, *Messor nuvia***nom. nov.**, *Pheidole arnoldi qindra***nom. nov.**, *Pseudoneoponera sublaevis quorra***nom. nov.**, *Strumigenys wylixa***nom. nov.**, *Camponotus foleyi cybira***nom. nov.**, *Camponotus maculatus brexyl***nom. nov.**, *Camponotus nixra***nom. nov.**, *Camponotus planus brina***nom. nov.**, *Carebara dazia***nom. nov.**, *Carebara xynera***nom. nov.**, *Colobopsis vitrea yevira***nom. nov.**, *Formica draven***nom. nov.**, *Messor draxil***nom. nov.**, *Pheidole singaporensis zolara***nom. nov.**, *Pseudolasius zynia***nom. nov.**, *Tetramorium drunex***nom. nov.**, *Tetramorium fenix***nom. nov.**, *Tetramorium flinex***nom. nov.**, *Temnothorax fynor***nom. nov.**, and *Tetraponera vantix***nom. nov.** In addition, *Estevia***nom. nov.** is proposed for the preoccupied genus *Wilsonia* Hong, 2002. Additional taxonomic changes are presented, including the reinstatement of *Tetramorium schultzei* Forel, 1910a **stat. nov.** and the following new combinations: *Bothroponera jonesii* (Forel, 1891), **comb. nov.**; *Diacamma unicolor* (Smith, 1860a), **comb. nov.**; *Pseudoneoponera vidua* (Smith, 1857), and *Diacamma solitaria* (Smith, 1860b), **comb. nov**.

## ﻿Introduction

A primary purpose of zoological nomenclature is to ensure that each taxon at every rank has a unique and stable name. In accordance with the International Code of Zoological Nomenclature (ICZN 1999), homonyms — identical names for different taxa at the same rank, whether within the species group, genus group, or family group — must be resolved to maintain this stability. When two taxa share the same name, the later (junior) homonym must either be replaced by an available synonym or, if no appropriate synonym exists, by a new replacement name (nomen novum) (ICZN Article 60).

Homonymy is particularly problematic in ants (Formicidae), a group with a long history of taxonomic revisions and a high diversity of described species. Currently, 16,881 valid species and subspecies of ants are recognized worldwide (AntCat 2025), reflecting more than 250 years of continuous taxonomic work beginning with Linnaeus (1758). The recurrent use of common descriptive epithets (e.g., *niger*, *rufus*, *elongatus*), compounded by limited historical access to prior literature, has led to the creation of multiple junior homonyms. Additionally, generic boundaries that have shifted over time have resulted in secondary homonyms as species are moved between genera.

The consequences of unresolved homonymy go beyond nomenclatural confusion. Unresolved homonymy can lead to misinterpretations in biodiversity data integration and conservation planning, especially in the era of big data (Morrison et al. 2009; Patterson et al. 2010; Sánchez-González 2020).

This review seeks to provide clarity by examining ICZN guidelines and illustrating their application through historical and modern examples within Formicidae. In doing so, I hope to provide guidelines and historical context for ant taxonomists. Overall, this work takes a practical approach that emphasizes avoiding unnecessary nomenclatural changes while ensuring that valid taxa are properly named and recognized.

### ﻿Simplified overview of ICZN rules for homonymy

The ICZN Code outlines strict rules to address homonymy. Two main categories are distinguished. Primary homonymy occurs when two taxa are originally described under the same genus using the same species-group name. For example, if two species were named “Formica rufa” by different authors, the latter-named one is a junior primary homonym and must be replaced. According to Article 57.2, junior primary homonyms are permanently invalid and cannot be used, regardless of later taxonomic changes.

Secondary homonymy arises when species described in different genera are later placed in the same genus, resulting in identical combinations. In such cases, the junior name becomes invalid under Article 59.1, but only if the taxa are considered congeneric. If subsequent revisions treat the species as belonging to separate genera and they have not been regarded as congeneric since 1899, the junior name may be maintained (Article 23.9.5).

A replacement name (nomen novum) should only be proposed if the junior homonym is an accepted name in use and no available synonym exists (ICZN Articles 23.3.5, 60.3); this name will then compete for priority with any synonym recognized later. If a junior homonym was already replaced by a nomen novum prior to 1961 and that replacement name is still in use, the replacement name retains priority, even if the act was unnecessary (ICZN Article 59.3). These provisions are intended to prevent the proliferation of superfluous names while promoting clarity and stability in nomenclature.

### ﻿Practical considerations

Addressing homonymy requires careful attention to historical taxonomy, synonymy, and the ICZN rules. Before introducing a new name, taxonomists should undertake a thorough review of the nomenclatural history of both the senior and junior homonyms. This includes verifying type specimens (AntWeb 2025), consulting original descriptions, and reviewing synonymies.

Modern tools such as AntCat.org (https://antcat.org/) and ZooBank (https://zoobank.org/) provide critical resources for checking name availability and synonym status. However, even with digital tools, homonym detection and resolution remain challenging, particularly in groups with extensive historical literature.

As emphasized by Gardner and Hayssen (2004), replacement names should be introduced with precision and only when truly necessary. Thorough historical research and careful verification of original sources are required to avoid introducing unnecessary new names, which can further complicate taxonomic databases and scientific communication. When in doubt, authors should err on the side of nomenclatural stability and avoid coining new names if an existing synonym or classification solution is available. Neubauer et al. (2014) illustrate this restrained approach in proposing replacement of homonyms within European freshwater gastropods. Those authors documented six cases of species-group homonyms that were unambiguously considered junior synonyms, and thus they chose not to introduce new names for those taxa. This approach follows the spirit of stability. If a name is not needed (because the taxon is not valid or in use), introducing a new name would only add clutter. As those authors noted, the Code has no explicit instruction for disused homonyms, but “following the intent expressed in Article 23.9.5, which seems to discourage the proposal of unnecessary replacement names” they refrained from naming new replacements. In fact, the Code explicitly provides that such cases may be referred to the Commission for a ruling under its plenary power, and that until such a ruling is made, prevailing usage of both names should be maintained (ICZN 1999: Art. 82).

This nomenclatural review establishes criteria for proposing replacement names for homonyms, with an emphasis on stability and alignment with the ICZN Code. Replacement names are proposed only for junior homonyms (primary or secondary) that are accepted, currently in use, and lack available synonyms. For primary homonyms, a replacement is introduced only when the taxa have been regarded as congeneric since 1899 (Art. 23.9.5). For secondary homonyms, a replacement is proposed only when the taxon occupies a stable generic placement supported by molecular or strong taxonomic evidence, consistent with the Code’s emphasis on nomenclatural stability (Art. 23.9.5). Although all junior primary homonyms are technically invalid under the Code, replacement names are introduced only for those that are currently accepted taxa, not for names that are junior synonyms or otherwise not in use. For example, Suppl. material 1 lists 155 unresolved junior primary homonyms among ant taxa that are treated as synonyms or are taxonomically indeterminate; for these, no replacement names are introduced, since doing so would not contribute to stability. In all cases, a complete historical review of taxonomic combinations is essential before any replacement is made.

Replacement names proposed in this work were selected to ensure uniqueness and compliance with the ICZN, with priority given to names that are easy to use and phonetically distinct. While some replacement names in taxonomy preserve etymological intent, reference morphology, geography, or honor individuals, such semantic continuity is not required by the Code, and the primary goal in this work was to provide clear, stable, and unambiguous names.

### ﻿Type specimen, authorship, and date

Stability in nomenclature depends not only on name priority but also on accurately linking type specimens to accepted species-group names. Even in the absence of homonymy, this linkage can be complicated by the lack of standardized unique specimen identifiers in older taxonomic works. Homonyms exacerbate this problem, as illustrated below.

A nomen novum (replacement name) is attributed to the author who proposes it and dated to that publication. The original author of the junior homonym is not credited as the author of the replacement name. However, the nomen novum inherits the name-bearing type specimen of the replaced junior homonym. Thus, although the authorship is new, the type specimen of the nomen novum is that of the junior homonym it replaces. As a result, the nomen novum becomes a senior objective synonym of the replaced name, since it is based on the same type (ICZN Article 60.1). A change in type is permitted only under exceptional circumstances, such as when the original type is inadequate or non-diagnostic following Recommendation 60A (e.g., as described in Article 75.5 or, in the case of a genus-group homonym, the type species is poorly defined).

If the junior homonym is replaced not with a nomen novum but with an existing synonym, then the type specimen(s) of that synonym becomes the name-bearing type(s) of the accepted taxon. Nevertheless, the type specimen of the junior homonym remains important, as synonymy is reversible. Should taxonomic revisions restore the junior homonym to validity, its type specimen will again carry nomenclatural weight. This creates a rare scenario where two distinct type specimens, those of the accepted synonym and of the suppressed homonym, must be tracked and curated.

To reduce future instances of homonymy and their attendant complexities, Formicidae taxonomists are strongly encouraged to propose species-group names that are unique across the family. While the ICZN permits identical species epithets in different genera, the practical burden of resolving homonyms is considerable for modern taxonomic databases and digital biodiversity platforms. Avoiding homonyms from the outset enhances long-term nomenclatural clarity and database integrity.

### ﻿Case studies in Formicidae

Within Formicidae, 97 instances were identified where previously proposed replacement names are now unnecessary. In most cases, these names were rendered superfluous when a secondary junior homonym was no longer considered congeneric with its senior homonym, thus eliminating the homonymy. In other cases, replacement names were unnecessary because an available synonym already existed and could serve as the valid name. Finally, several new names were introduced due to oversight, even though valid replacement names had already been established.

The following case studies illustrate common pitfalls and resolutions in the management of homonymy within ants. By highlighting these patterns, I aim to reduce the future creation of homonyms and to guide best practices when addressing unresolved cases, particularly by avoiding unnecessary nomina nova. Each case is presented in the context of the relevant ICZN Articles, underscoring the importance of a careful, context-aware taxonomic approach.

All names in the case studies are provided in both their original combination (protonym) and, where applicable, their current valid combination.

#### ﻿Temporary nature of secondary homonymy

Secondary homonyms may be temporary due to shifting genus boundaries. Proposing replacement names in such cases has often proved premature. It is preferable not to propose a replacement name until generic limits stabilize. This is especially relevant in ants, where molecular phylogenetics and, more recently, phylogenomic datasets (e.g., Ward et al. 2015; Branstetter et al. 2017; Blaimer et al. 2018; Doré et al. 2025) have led to substantial redefinitions of genera and higher-level relationships. While these studies have greatly improved our understanding of evolutionary lineages, they also highlight the fluidity of generic boundaries in some groups. Until phylogenomic frameworks are more fully integrated and stabilized across all Formicidae genera, restraint in proposing replacement names for secondary homonyms is warranted.

Case 1

*Micromyrma pygmaea* Dufour, 1857: 61 (current combination *Tapinoma pygmaeum*) was combined in *Tapinoma* in 1859.

*Formica pygmaea* Latreille, 1798: 45 was combined in *Tapinoma* in 1852 and then *Plagiolepis* in 1861.

*Tapinoma dufouri* Donisthorpe, 1943: 662 was proposed as a replacement name for *Tapinoma pygmaeum* Dufour, 1857: 61, but is unnecessary as *Tapinoma pygmaeum* Dufour, 1857: 61 was no longer a secondary junior homonym.

Case 2

Meanwhile, *Micromyrma dufourii* Perris, 1878: 382 (current combination *Plagiolepis dufourii*) was combined in *Tapinoma* in 1983 but moved to *Plagiolepis* in 1925. But that did not stop *Tapinoma confusum* Smith, 1951: 837 being proposed as a replacement name for *Tapinoma dufouri* Donisthorpe, 1943: 662.

Case 3

When described, *Ponera apicalis* Smith, 1857: 66 (current combination *Ectomomyrmex apicalis*) was a junior secondary homonym of *Formica apicalis* Latreille, 1802: 204 (current combination *Neoponera apicalis*) which was combined in *Ponera* by Latreille, 1809: 128. However, Mayr (1863: 439) combined the taxa in *Pachycondyla* in 1863. Thus, *Ponera apicalis* Smith, 1857: 66 was a junior secondary homonym in *Ponera* from 1857–1863. By the time *Ectomomyrmex terminalis* Smith, 1871: 321 was proposed as a replacement name, the name was no longer needed. *Ectomomyrmex terminalis* Smith, 1871: 321 is an unnecessary replacement name and an objective synonym of *Ponera apicalis* Smith, 1857: 66.

For *Ponera apicalis* Smith, 1857: 66, the secondary homonym story continues. *Ponera apicalis* Smith, 1857: 66 was combined in *Pachycondyla* by Brown in 1995 (Brown 1995: 302), joining *Formica apicalis* Latreille, 1802: 204. Thus, once again, *Ponera apicalis* Smith, 1857: 66 was a junior secondary homonym. In 2010, homonym hunter Özdikmen created the replacement name *Ectomomyrmex terminalis* Özdikmen, 2010: 993 for *Ponera apicalis* Smith, 1857: 66. However, in 2014, both *Ponera apicalis* Smith, 1857: 66 and *Formica apicalis* Latreille, 1802: 204 left *Pachycondyla* for different genera (Schmidt and Shattuck 2014). This shift made *Ectomomyrmex terminalis* Özdikmen, 2010: 993 an unnecessary name in 2014 and an objective synonym of *Ponera apicalis* Smith, 1857: 66.

This sequence illustrates the risk of proposing new names based on transient classifications. Another issue is Özdikmen’s (2010) use of the same species epithet proposed by Smith, 1871. Replacement names, even when unnecessary, still need to be recorded and managed, the proposal of an identical name to replace a homonym does not help. Identical names should never be proposed.

Case 4

*Wheeleria santschii* Forel, 1905: 171 (current combination *Monomorium santschii*).

In 1906, Forel (1906: 64) noted that the genus-group name *Wheeleria* was already in use and thus a junior homonym of *Wheeleria* Tutt, 1905: 37 (Lepidoptera). Forel was correct to provide a genus replacement name *Santschia* Forel, 1906: 65. However, Forel also unnecessarily proposed a replacement name for the species *Santschia intrudens* Forel, 1906: 65 [perhaps to avoid having the genus and species names to be the nearly identical *Santschia santschii* (Forel 1905: 171).] However, the replacement name is unnecessary, and thus, *Monomorium intrudens* Forel, 1906: 65, is now an objective junior synonym of *Monomorium santschii* Forel, 1905: 171. In addition, the proposed name is a junior secondary homonym of *Monomorium intrudens* Smith, 1874: 406. Because there is an existing, valid synonym for *Monomorium intrudens* Forel, 1906: 65, the replacement name for *Monomorium intrudens* Forel, 1906: 65 is *Monomorium santschii* Forel, 1905: 171.

#### ﻿Junior secondary homonym replaced before 1961

If a junior secondary homonym was replaced before 1961, and the substitute name remains in use, it retains priority under ICZN Article 59.3. The following case illustrates this problem:

*Syllophopsis jonesi* Arnold, 1952: 465 was proposed as a replacement name for *Syllophopsis arnoldi* Santschi, 1921: 120; a junior secondary homonym of *Monomorium arnoldi* Forel, 1913a: 137. The replacement name *Syllophopsis jonesi* Arnold, 1952: 465 was proposed before 1961 (when both *arnoldi* taxa were included in *Monomorium*). Article 59.3 stipulates that valid pre-1961 replacement names retain priority, even if the original homonymy is later resolved. In 2015, Ward et al. combined *arnoldi* Santschi, 1921: 120 in *Syllophopsis* and mistakenly proposed that *jonesi* was an unnecessary replacement name. The priority awarded by Ward et al. (2015: 73) is incorrect and *Syllophopsis jonesi* Arnold, 1952: 465 has priority.

#### ﻿Complete taxonomic histories required

Confusion also arises when earlier replacements are overlooked; the following case illustrates this problem.

*Anochetus jacobsoni* Menozzi, 1939: 178 is a junior primary homonym of *Anochetus jacobsoni* Forel, 1911b: 193. *Anochetus menozzii* Donisthorpe, 1941b: 237 was proposed as a replacement name. This new name was overlooked and a second and unnecessary replacement name was proposed: *Anochetus ineditus* Baroni Urbani, 1971: 360.

#### ﻿Use of available synonyms

If an available synonym exists for the junior secondary homonym, the synonym should be used instead of proposing a new name. However, a replacement name has priority if proposed before a junior synonym.

Case 1

*Ponera simillima* Smith, 1860b: 105 (current combination *Prionopelta simillima*) is a junior primary homonym of *Leptogenys simillima* Smith, 1860b: 104 (original combination: *Ponera simillima*). [The same species epithet was used one page later to describe a different species]. *Prionopelta nominata* Smith, 1871: 323 was proposed as a replacement name for the junior homonym but this name was overlooked, and a second replacement name was proposed, *Prionopelta poultoni* Donisthorpe, 1932: 462. Subsequently Brown (1953: 12) synonymized three species, including *Prionopelta majuscula* Emery, 1897: 595 with *Ponera simillima* Smith, 1860b: 105, and thought the oldest synonym *Prionopelta majuscula* Emery, 1897: 595 would take priority as a replacement name for *Ponera simillima* Smith, 1860b: 105. However, nom. nov. proposed before the synonymy take priority, and thus *Prionopelta nominata* Smith, 1871: 323, nom. restit. has priority as the replacement name.

Case 2

Here is another example of an overlooked replacement name and the use of a synonym that occurred after the nomen novum replacement name. All species are currently in *Anochetus*, but I use original combinations to help keep the story straight.

*Odontomachus tyrannicus* Smith, 1861: 44 is a junior primary homonym of *Odontomachus tyrannicus* Smith, 1859: 144. As with the *Ponera simillima* example above, we have name duplication by the same author within the same genus. The situation is further complicated: Smith had shared specimens with colleagues and labeled them “gladiator” (see Brown 1978: 574, suggesting that *gladiator* was the intended species epithet for *Odontomachus tyrannicus* Smith, 1861: 44). In 1862, Mayr included one of the specimens labeled *gladiator* in the description of a new genus for the taxon and used *gladiator* as the species name, providing an unintentional but formal description of the new species *Stenomyrmex gladiator* Mayr, 1862: 712.

In 1863, Roger proposed *Odontomachus smithii* Roger, 1863: 21 as a replacement name for *Odontomachus tyrannicus* Smith, 1861: 44.

In 1871, *Odontomachus gladiator* Smith, 1871: 320 was proposed as a replacement name.

In 1893, *Stenomyrmex gladiator* Mayr, 1862: 712 was proposed as a junior synonym of *Odontomachus tyrannicus* Smith, 1861: 44 by Dalla Torre 1893: 48.

In 1932, *Odontomachus gladiator* Donisthorpe, 1932: 467 was proposed as a replacement name for *Odontomachus tyrannicus* Smith, 1861: 44.

Brown (1978: 556) mistakenly prioritizes *Stenomyrmex gladiator* Mayr, 1862: 712 as the replacement name, although *Odontomachus smithii* Roger, 1863: 21 nom. restit. actually has priority.

## ﻿Results

In the homonym lists below, collections are referred to by the following acronyms. When the type depository is not known and not given in the original description, it is recorded here as “unspecified”.

**AMNH**American Museum of Natural History, New York, USA;

**ANIC**Australian National Insect Collection, Canberra, Australia;

**ATPC** Alberto Tinaut personal collection, Universidad de Granada, Granada, Spain;

**CASC**California Academy of Sciences, San Francisco, USA;

**DEIB**Deutsches Entomologisches Institut, Berlin-Dahlem, Germany;

**HNHM**Hungarian Natural History Museum, Budapest, Hungary;

**IEUB**Istituto di Entomologia “Guido Grandi”, Università degli Studi di Bologna, Bologna, Italy;

**LACM**Los Angeles County Museum, Los Angeles, USA;

**LKCNHM** Lee Kong Chian Natural History Museum, Singapore;

**MCZC**Museum of Comparative Zoology, Harvard University, Cambridge, USA;

**MHNG**Muséum d’histoire naturelle, Geneva;

**MNHN**Muséum National d’Histoire Naturelle, Paris;

**MNHU**Museum für Naturkunde an der Humboldt-Universität zu Berlin (Zoologisches Museum), Berlin, Germany;

**MSNG**Museo Civico di Storia Naturale “Giacomo Doria”, Genoa, Italy;

**MVMA** Museum of Victoria, Melbourne, Victoria, Australia;

**NHMB**Naturhistorisches Museum Basel, Basel, Switzerland;

**NHMUK**British Museum of Natural History, now Natural History Museum London, London, England;

**NHMW**Naturhistorisches Museum Wien, Vienna, Austria;

**NUYC** Laboratory of Insect Specimens, Agricultural College, Ningxia University, Yinchuan, China;

**OXUM**Oxford University Museum of Natural History, Oxford, England;

**SAMC**Iziko Museums of Cape Town (= South African Museum), Cape Town, South Africa;

**SIZK**Schmalhausen Institute of Zoology, Kyiv, Ukraine;

**SMNG**Senckenberg Museum für Naturkunde (Staatliches Museum für Naturkunde), Görlitz, Germany;

**SWFU**Faculty of Conservation Biology, Southwest Forestry College [University], Kunming, Yunnan, China;

**WUIK** Wonkwang University, Division of Biological Sciences, Iksan (= Iri City), North Cholla Province, Republic of Korea.

### ﻿A. Genus-group name homonyms

#### ﻿ Estevia

Taxon classificationAnimaliaHymenopteraFormicidae

Fisher nom. nov.

9509854B-8623-5897-8CEC-6EE17610592F

† Wilsonia Hong, 2002: 608 (Insecta, Hymenoptera). Preoccupied by Wilsonia Bonaparte, 1838: 23 (Aves).

##### Type species.

†*Wilsonia megagastrosa* Hong, 2002: 608, by original designation.

##### Etymology.

The genus name is proposed in honor of Flávia Esteves, a Brazilian myrmecologist and taxonomist, recognized for her significant contributions to the study of ant morphology, diversity, and systematics. Gender: feminine.

#### ﻿ Estevia megagastrosa

Taxon classificationAnimaliaHymenopteraFormicidae

Hong, 2002 comb. nov.

4F7F7FC2-5C1C-5FA4-B5A1-1AF87EEC20A2

†Wilsonia megagastrosa Hong, 2002: 608, figs 565–573 (w.) China (Liaoning, Fushun Amber, Eocene).

### ﻿B. Primary homonyms

Below are the primary homonyms that satisfy the following criteria for the creation of a new replacement name (nomen novum): the junior primary homonym is an accepted taxon, that is, in use as a valid species. In one case, the senior homonym is not valid (a junior synonym of another species) but a new name is still proposed since the junior homonym is in use.

Replacement names are not established when the junior primary homonym is a disused junior synonym (and already treated as a synonym), when the junior primary homonym’s status is highly doubtful, nor when the two homonyms have not been congeneric for over 100 years. All such long-separated homonym pairs are listed in Section C (ICZN Article 23.9.5), where no replacement names are introduced and prevailing usage is maintained.

Homonyms are presented first with the original combination and then the current combination is given if different than the original combination. Homonyms are listed by the current combination of the junior primary homonym.

#### ﻿B1. Acromyrmex atrina

Taxon classificationAnimaliaHymenopteraFormicidae

Fisher nom. nov.

5839B14C-FD38-5F0A-838C-D095914B6633

##### Note.

Replacement name for Atta (Acromyrmex) subterranea var. brunnea Forel, 1912b: 181 (current combination *Acromyrmex brunneus*), junior primary homonym of *Atta brunnea* Patton, 1894: 618 (current combination *Odontomachus brunneus*).

##### Type locality.

“Palmeiras, Prov. Rio, Brésil (Goeldi); São Paulo (Brésil); Ceara, Brésil (Diaz da Rocha); Teixeira Suares, Brésil (Sampaio)” [Palmeiras, Rio de Janeiro State; São Paulo State; Ceará State; Teixeira Soares, Paraná State, Brazil].

##### Type material.

Syntype workers.

##### Type depository.

MHNG.

##### Etymology.

Derived from Latin *ater*, *atra*, *atrum* (adjective for black, dark). The form atrina is treated here as a noun in apposition, and is therefore indeclinable with respect to the gender of the genus.

#### ﻿B2. Camponotus dorex

Taxon classificationAnimaliaHymenopteraFormicidae

Fisher nom. nov.

836F54B1-D3C4-5FF5-859F-C96E18498CE8

##### Note.

Replacement name for *Camponotus concavus* Kim & Kim, 1994: 285, junior primary homonym of Camponotus aethiops var. concava Dalla Torre, 1893, junior synonym of *Camponotus aethiops* (Latreille, 1798: 35).

##### Type locality.

South Korea: Jeonnam, Paekyang Temple.

##### Type material.

Holotype worker.

##### Type depository.

WUIK.

##### Etymology.

Arbitrary combination of letters, treated as a noun in apposition.

#### ﻿B3. Camponotus durnyx

Taxon classificationAnimaliaHymenopteraFormicidae

Fisher nom. nov.

EBB79CB8-F92E-5995-AB9F-45C4664FC426

##### Note.

Replacement name for *Camponotus horni* Clark, 1930: 308, junior primary homonym of *Camponotus horni* Kirby, 1896: 58 (current combination *Iridomyrmex horni*), junior synonym of *Iridomyrmex purpureus* (Smith, 1858).

##### Type locality.

Australia: Northern Territory, Palm Creek.

##### Type material.

Holotype queen.

##### Type depository.

MVMA.

##### Etymology.

Arbitrary combination of letters, treated as a noun in apposition.

#### ﻿B4. Camponotites flarex

Taxon classificationAnimaliaHymenopteraFormicidae

Fisher nom. nov.

EFCDB691-C0E9-5B1F-A7A1-E2A8310EF1A9

##### Note.

Replacement name for *Camponotus pictus*Zhang et al., 1994: 8 (fossil) (current combination *Camponotites pictus*), junior primary homonym of Camponotus ligniperdus var. pictus Forel, 1886, junior synonym of *Camponotus novaeboracensis* (Fitch, 1855).

##### Type locality.

China: Shanwang Basin (fossil locality, Miocene).

##### Type material.

Fossil imprint, registration number K0174 (original description did not specify an institutional repository; depository remains unknown). Validity is based on the published description and illustrations, which allow recognition of the taxon and justify replacement under ICZN Article 60.

##### Type depository.

Unspecified.

##### Etymology.

Arbitrary combination of letters, treated as a noun in apposition.

#### ﻿B5. Camponotus robecchii clyne

Taxon classificationAnimaliaHymenopteraFormicidae

Fisher nom. nov.

CB433489-3D2B-51D9-A644-4FA6E7827A75

##### Note.

Replacement name for Camponotus (Myrmotrema) troglodytes var. dispar Stitz, 1923: 165 (current combination *Camponotus robecchii dispar*), junior primary homonym of Camponotus siemsseni var. dispar Forel, 1902b: 287.

##### Type locality.

Namibia (“German Southwest Africa”): Kuibis.

##### Type material.

Syntype workers.

##### Type depository.

MNHU.

##### Etymology.

Arbitrary combination of letters, treated as a noun in apposition.

#### ﻿B6. Camponotus substitutus cylrix

Taxon classificationAnimaliaHymenopteraFormicidae

Fisher nom. nov.

41F41870-85BA-51EC-8566-785DFF3646D3

##### Note.

Replacement name for Camponotus (Tanaemyrmex) substitutus var. clara Stitz, 1938: 116, junior primary homonym of *Camponotus clarus* Mayr, 1862: 660.

##### Type locality.

Argentina.

##### Type material.

Syntype queens.

##### Type depository.

Unknown unspecified.

##### Etymology.

Arbitrary combination of letters, treated as a noun in apposition.

#### ﻿B7. Colobopsis breva

Taxon classificationAnimaliaHymenopteraFormicidae

Fisher nom. nov.

67DB4D55-2FE5-5663-8064-2C3C9BF1AB85

##### Note.

Replacement name for Camponotus (Colobopsis) vitreus var. latinota Stitz, 1925: 127 (current combination *Colobopsis vitrea latinota*), junior primary homonym of Camponotus foraminosus subsp. latinotus Forel, 1907b: 144, junior synonym of *Camponotus galla* Forel, 1894.

##### Type locality.

Philippines: Mindanao.

##### Type material.

Syntype queens.

##### Type depository.

MNHU.

##### Etymology.

Arbitrary combination of letters, treated as a noun in apposition.

#### ﻿B8. Crematogaster scutellaris gynia

Taxon classificationAnimaliaHymenopteraFormicidae

Fisher nom. nov.

CEABBABB-FED8-590F-BF81-F63E2833F6EC

##### Note.

Replacement name for Crematogaster scutellaris var. nigra Krausse, 1912: 164, junior primary homonym of Crematogaster chiarinii var. nigrum Forel, 1910b: 434.

##### Type locality.

Italy: Sardinia, Asuni.

##### Type material.

Syntype workers.

##### Type depository.

MSNG.

##### Etymology.

Arbitrary combination of letters, treated as a noun in apposition.

#### ﻿B9. Dolichoderus thoracicus jexina

Taxon classificationAnimaliaHymenopteraFormicidae

Fisher nom. nov.

E5EBFB40-5F88-5CD7-8159-5774F363BA8E

##### Note.

Replacement name for Dolichoderus (Hypoclinea) bituberculatus var. rufescens Stitz, 1925: 123 (current combination *Dolichoderus thoracicus rufescens*), junior primary homonym of Dolichoderus (Monacis) debilis var. rufescens Mann, 1912: 40 (current combination *Dolichoderus rufescens*).

##### Type locality.

Borneo.

##### Type material.

Syntype workers.

##### Type depository.

MNHU.

##### Etymology.

Arbitrary combination of letters, treated as a noun in apposition.

#### ﻿B10. Ectomomyrmex pylixy

Taxon classificationAnimaliaHymenopteraFormicidae

Fisher nom. nov.

10DF3557-B0C1-5C73-8C21-3FA7D98324BF

##### Note.

Replacement name for Pachycondyla (Ectomomyrmex) punctata Karavaiev, 1935: 71 (current combination *Ectomomyrmex punctatus*), unresolved junior primary homonym of *Pachycondyla punctata* Smith, 1858: 108 (current combination *Platythyrea punctata*).

The replacement name uses the same type specimen and type locality originally identified for the name replaced.

##### Type locality.

Vietnam (“Zentralannam”): Bana.

##### Type material.

Syntype workers, syntype queens.

##### Type depository.

SIZK.

##### Etymology.

Arbitrary combination of letters, treated as a noun in apposition.

#### ﻿B11. Ectomomyrmex pylor

Taxon classificationAnimaliaHymenopteraFormicidae

Fisher nom. nov.

81331E14-4498-5681-B85F-4638D4E64662

##### Note.

Replacement name for Pachycondyla (Ectomomyrmex) sauteri Forel, 1912a: 49 (current combination *Ectomomyrmex sauteri*), unresolved junior primary homonym of Pachycondyla (Pseudoponera) sauteri Wheeler, 1906: 304 (current combination *Cryptopone sauteri*).

The replacement name uses the same type specimen and type locality originally identified for the name replaced.

##### Type locality.

Taiwan (“Formosa”): Pilam.

##### Type material.

Syntype workers, syntype queens.

##### Type depository.

MHNG.

##### Etymology.

Arbitrary combination of letters, treated as a noun in apposition.

#### ﻿B12. Euponera tynara

Taxon classificationAnimaliaHymenopteraFormicidae

Fisher nom. nov.

3A6FD4ED-F66F-5819-81CA-9DB92C67B949

##### Note.

Replacement name for *Ponera grandis* Donisthorpe, 1947: 283 (current combination *Euponera grandis*), unresolved junior primary homonym of *Ponera grandis* Guérin-Méneville, 1838: 206 (current combination *Dinoponera grandis*).

The replacement name uses the same type specimen and type locality originally identified for the name replaced.

##### Type locality.

Vietnam: Tonkin, Yen Bay.

##### Type material.

Holotype worker.

##### Type depository.

NHMUK.

##### Etymology.

Arbitrary combination of letters, treated as a noun in apposition.

#### ﻿B13. Formica lavateri liora

Taxon classificationAnimaliaHymenopteraFormicidae

Fisher nom. nov.

0B1DCCED-433B-5AD0-93FE-C6A0499BBB86

##### Note.

Replacement name for Formica lavateri var. major Heer, 1867: 11, junior primary homonym of *Formica major* Nylander, 1849: 29, junior synonym of *Formica polyctena* Foerster, 1850: 15.

##### Type locality.

Croatia (Miocene).

##### Type material.

Fossil imprints (original description did not specify an institutional repository; Heer’s fossil material is generally housed in the Paläontologisches Institut und Museum der Universität Zürich, but the depository for this specimen is not confirmed).

##### Type depository.

Unspecified.

##### Etymology.

Arbitrary combination of letters, treated as a noun in apposition.

#### ﻿B14. Formica mytara

Taxon classificationAnimaliaHymenopteraFormicidae

Fisher nom. nov.

1C6800A5-BA70-5D31-975F-57F4E4C91582

##### Note.

Replacement name for *Formica robusta* Chang & He, 2002: 53, junior primary homonym of *Formica robusta* Carpenter, 1930: 56.

##### Type locality.

China: Ningxia Hui Autonomous Region, Liupan Mountains.

##### Type material.

Holotype worker.

##### Type depository.

NUYC.

##### Etymology.

Arbitrary combination of letters, treated as a noun in apposition.

#### ﻿B15. Hypoponera rylin

Taxon classificationAnimaliaHymenopteraFormicidae

Fisher nom. nov.

218EDBDC-36F5-5C20-9F17-DBF5216E39DF

##### Note.

Replacement name for *Ponera gracilicornis* Menozzi, 1931: 266, junior primary homonym of *Ponera gracilicornis* Mayr, 1868: 72.

##### Type locality.

Costa Rica.

##### Type material.

Syntype workers, syntype queen(s).

##### Type depository.

DEIB, IEUB.

##### Etymology.

Arbitrary combination of letters, treated as a noun in apposition.

#### ﻿B16. Hypoponera veltan

Taxon classificationAnimaliaHymenopteraFormicidae

Fisher nom. nov.

493B3238-D849-592A-8F9C-E712123BC858

##### Note.

Replacement name for *Ponera lamellosa* Forel, 1907a: 4, junior primary homonym of *Ponera lamellosa* Roger, 1860: 295 (current combination *Platythyrea lamellosa*).

##### Type locality.

Malaysia: Malacca, Kuala Lumpur.

##### Type material.

Probable holotype worker (No indication of number of specimens is given).

##### Type depository.

HNHM.

##### Etymology.

Arbitrary combination of letters, treated as a noun in apposition.

#### ﻿B17. Lepisiota vynia

Taxon classificationAnimaliaHymenopteraFormicidae

Fisher nom. nov.

6445DC3D-BB47-5B58-996A-BAA6F9EAA435

##### Note.

Replacement name for Acantholepis monardi var. australis Santschi, 1930: 75 (current combination *Lepisiota monardi australis*), junior primary homonym of Acantholepis (Acrostigma) australis Forel, 1902c: 479 (current combination *Stigmacros australis*).

##### Type locality.

Angola: Tchitunda.

##### Type material.

Syntype workers.

##### Type depository.

NHMB.

##### Etymology.

Arbitrary combination of letters, treated as a noun in apposition.

#### ﻿B18. Temnothorax flavispinus novira

Taxon classificationAnimaliaHymenopteraFormicidae

Fisher nom. nov.

1E8C6FDB-A194-5544-A2E2-53344D7697A9

##### Note.

Replacement name for Leptothorax flavispinus subsp. crassispinus Cagniant, 1970: 421 (current combination *Temnothorax flavispinus crassispinus*), junior primary homonym of Leptothorax (Leptothorax) nylanderi var. crassispina Karavaiev, 1926: 69 (current combination *Temnothorax crassispinus*).

##### Type locality.

Tunisia: Dratamar, nr. Kairouan.

##### Type material.

Syntype workers.

##### Type depository.

NHMB.

##### Etymology.

Arbitrary combination of letters, treated as a noun in apposition.

#### ﻿B19. Messor minor nubina

Taxon classificationAnimaliaHymenopteraFormicidae

Fisher nom. nov.

9EB6C542-2EB2-5A4B-B742-FC8B19D0EF6F

##### Note.

Replacement name for Messor minor subsp. maurus Barquín, 1981: 81, junior primary homonym of Messor sublaeviceps var. maurus Santschi, 1927b: 244 (current combination *Messor picturatus maurus*).

##### Type locality.

Tunisia: Kairouan.

##### Type material.

Syntype workers, syntype queen(s).

##### Type depository.

NHMB.

##### Etymology.

Arbitrary combination of letters, treated as a noun in apposition.

#### ﻿B20. Messor nuvia

Taxon classificationAnimaliaHymenopteraFormicidae

Fisher nom. nov.

EA5184E4-A789-59A1-9DDE-AB3FFED407DF

##### Note.

Replacement name for *Messor testaceus* Bernard, 1980: 266, junior primary homonym of *Messor testaceus* Donisthorpe, 1950: 68.

##### Type locality.

Syria.

##### Type material.

Holotype worker.

##### Type depository.

MNHN.

##### Etymology.

Arbitrary combination of letters, treated as a noun in apposition.

#### ﻿B21. Pheidole arnoldi qindra

Taxon classificationAnimaliaHymenopteraFormicidae

Fisher nom. nov.

341AE1FC-F647-512A-890C-44BD0A99097B

##### Note.

Replacement name for Pheidole arnoldi var. rufescens Arnold, 1920: 449, junior primary homonym of Pheidole sitarches subsp. rufescens Wheeler, 1908: 443.

##### Type locality.

Zimbabwe (“S Rhodesia”): Sipapoma.

##### Type material.

Syntype workers.

##### Type depository.

NHMUK, SAMC.

##### Etymology.

Arbitrary combination of letters, treated as a noun in apposition.

#### ﻿B22. Pseudoneoponera sublaevis quorra

Taxon classificationAnimaliaHymenopteraFormicidae

Fisher nom. nov.

1444206B-7121-5AF0-91A6-AED7DDF3AFCB

##### Note.

Replacement name for Ponera (Bothroponera) sublaevis r. reticulata Forel, 1900a: 62 (current combination *Pseudoneoponera sublaevis reticulata*), junior primary homonym of *Ponera reticulata* Smith, 1858: 85 (current combination *Odontoponera reticulata*).

##### Type locality.

Australia: Queensland.

##### Type material.

Syntype workers, syntype males.

##### Type depository.

ANIC, MHNG.

##### Etymology.

Arbitrary combination of letters, treated as a noun in apposition.

#### ﻿B23. Strumigenys wylixa

Taxon classificationAnimaliaHymenopteraFormicidae

Fisher nom. nov.

A84C804F-BE9F-5C50-8419-14371BD0639C

##### Note.

Replacement name for *Strumigenys intermedia* Tang & Guénard, 2023: 68, junior primary homonym of Strumigenys alberti var. intermedia Wheeler, 1913: 242, junior synonym of *Strumigenys alberti* Forel, 1893: 380.

##### Type locality.

China: Hong Kong, Lantau Island.

##### Type material.

Holotype worker.

##### Type depository.

LKCNHM.

##### Etymology.

Arbitrary combination of letters, treated as a noun in apposition.

### ﻿C. ICZN Article 23.9.5

The seven names listed below are not assigned replacement names, in accordance with ICZN Article 23.9.5. When an author discovers that a species-group name currently in use is a junior primary homonym (Art. 53.3) of another species-group name also in use, but the two names apply to taxa not considered congeneric after 1899, the junior homonym should not be automatically replaced. Instead, the case should be referred to the Commission for a ruling under its plenary power. In the meantime, prevailing usage of both names is to be maintained (Art. 82).

**C1.** No replacement name for *Formica badia* Smith, 1857: 54 (combination in *Camponotus* by Roger (1863: 3), current combination *Colobopsis badia*), junior primary homonym of *Formica badia* Latreille, 1802: 238 (combination in *Atta* by Lepeletier de Saint-Fargeau (1835: 174), current combination *Pogonomyrmex badius*).

**Type locality.** Singapore.

**Type material.** Lectotype.

**Type depository.**OXUM.

**Note.** Primary homonyms have not been congeneric since 1835.

**C2.** No replacement name for *Formica cordata* Smith, 1859: 137 (combination in *Hypoclinea* by Mayr (1879: 659), current combination *Philidris cordata*), junior primary homonym of *Formica cordata* Holl, 1829: 140 (combination in *Pheidole* by Mayr (1868: 17), Boudinot et al. (2024: 148), current combination *Pheidole cordata*).

**Type locality.** Indonesia: Aru Islands.

**Type material.** Syntype workers.

**Type depository.**OXUM.

**Note.** Primary homonyms have not been congeneric since 1868.

**C3.** No replacement name for *Formica nigerrima* Nylander, 1856: 71 (combination in *Tapinoma* by Mayr (1861: 41), current combination *Tapinoma nigerrimum*), junior primary homonym of *Formica nigerrima* Christ, 1791: 513 (combination in *Lasius* by Emery (1892a: 162), current combination *Lasius nigerrimus*).

**Type locality.** France: Prades-le-Lez.

**Type material.** Neotype worker.

**Type depository.**SMNG.

**Note.** Primary homonyms have not been congeneric since 1861.

**C4.** No replacement name for *Formica nitida* Smith, 1859: 138 (combination in *Camponotus* by Roger (1863: 4), current combination *Camponotus nitidus*), junior primary homonym of *Formica nitida* Razoumowsky, 1789: 300.

**Type locality.** Indonesia: Aru.

**Type material.** Holotype worker.

**Type depository.**OXUM.

**Note.** Primary homonyms have not been congeneric since 1863.

**C5.** No replacement name for *Formica pallida* Smith, 1857: 57 (combination in *Camponotus* by Mayr (1862: 656), current combination *Camponotus irritans pallidus*), junior primary homonym of *Formica pallida* Latreille, 1798: 41 (combination in *Lasius* by Emery (1893: 183), current combination *Lasius pallida*), junior synonym of *Lasius brunneus* (Latreille, 1798: 41).

**Type locality.** Malaysia: Borneo, Sarawak.

**Type material.** Syntype workers.

**Type depository.**NHMUK.

**Note.** Primary homonyms have not been congeneric since 1862.

**C6.** No replacement name for *Formica timida* Jerdon, 1851: 122 (combination in *Camponotus* by Roger (1863: 3), current combination *Camponotus timidus*), junior primary homonym of *Formica timida* Foerster, 1850: 35 (combination in *Lasius* by Nylander (1856: 68), current combination *Lasius timida*), junior synonym of *Lasius brunneus* (Latreille, 1798: 41).

**Type locality.** India: Malabar coast.

**Type material.** Syntype workers, syntype queen.

**Type depository.** Unspecified.

**Note.** Primary homonyms have not been congeneric since 1862.

**C7.** No replacement name for *Ponera sulcata* Mayr, 1867: 441 (combination in Pachycondyla (Bothroponera) by Emery (1901a: 46), current combination *Bothroponera sulcata*), unresolved junior primary homonym of *Ponera sulcata* Smith, 1858: 99 (combination in Ectatomma (Gnamptogenys) by Emery (1893: 26), current combination *Gnamptogenys sulcata*).

**Type locality.** India. [Emery, 1911: 78 gives India as type locality, possibly based on the collection data in Forel 1900b: 326, and Bingham 1903: 99.]

**Type material.** Syntype workers.

**Type depository.**NHMW.

**Current subspecies** (nominal subspecies plus the following):

﻿*B. sulcata fossulata* (Forel, 1900b: 323)

﻿*B. sulcata sulcatotesserinoda* (Forel, 1900b: 323).

**Note.** Primary homonyms have not been congeneric since 1893.

### ﻿D. Secondary homonyms

Under ICZN Article 59.1, a junior secondary homonym must be replaced if both names are in use and are congeneric. The following cases satisfy the criteria for the creation of a new replacement name (nomen novum): the junior secondary homonym is an accepted taxon, that is, in use as a valid species. Replacement names are not established when the junior secondary homonym is a disused junior synonym (and treated as a synonym already), when the junior secondary homonym’s status is highly doubtful, nor when the secondary homonym generic placement is not certain (see section E for a list of these exceptions).

#### ﻿D1. Camponotus chucki

Taxon classificationAnimaliaHymenopteraFormicidae

Fisher nom. nov.

EB9FA6EE-B3BF-5835-8DE7-2E570A8ADA1C

##### Note.

Replacement name for *Camponotus kugleri* Mackay, 2025: 453, junior secondary homonym of *Camponotus kugleri* Ionescu-Hirsch, 2010: 78.

##### Type locality.

Colombia: Zona Buenos Aires.

##### Type material.

Holotype minor worker.

##### Type depository.

MCZC.

##### Etymology.

The name honors Charles (“Chuck”) Kugler, myrmecologist and Peace Corps colleague, who collected the holotype and numerous specimens of *Camponotus* in Colombia. The epithet is treated as a noun in apposition.

#### ﻿D2. Camponotus foleyi cybira

Taxon classificationAnimaliaHymenopteraFormicidae

Fisher nom. nov.

DBD9C8DC-01D4-5578-8E0F-8FDDF6834704

##### Note.

Replacement name for Camponotus (Tanaemyrmex) foleyi subsp. rufescens Bernard, 1953: 190, junior secondary homonym of Dendromyrmex fabricii var. rufescens Wheeler, 1916: 13 (current combination *Camponotus fabricii rufescens*), junior synonym of *Camponotus nidulans* (Smith, 1860c: 69).

##### Type locality.

Libya: Fezzân, El Abiod.

##### Type material.

Syntype major workers.

##### Type depository.

MNHN.

##### Etymology.

Arbitrary combination of letters, treated as a noun in apposition.

#### ﻿D3. Camponotus maculatus brexyl

Taxon classificationAnimaliaHymenopteraFormicidae

Fisher nom. nov.

C7C273B9-058A-55AF-8428-34BB4DC73F7B

##### Note.

Replacement name for Camponotus (Myrmoturba) maculatus r. foveolatus Stitz, 1925: 125, junior secondary homonym of *Formica foveolata* Mayr, 1853: 277 (current combination *Camponotus lateralis foveolatus*, junior synonym of *Camponotus piceus* (Leach, 1825: 292).

##### Type locality.

Philippines.

##### Type material.

Syntype workers.

##### Type depository.

MNHU.

##### Etymology.

Arbitrary combination of letters, treated as a noun in apposition.

#### ﻿D4. Camponotus nixra

Taxon classificationAnimaliaHymenopteraFormicidae

Fisher nom. nov.

7F7F4F76-C34E-5DB2-AF30-6BE26AF4DFA2

##### Note.

Replacement name for Camponotus (Karavaievia) nigripes Dumpert, 1995: 96, junior secondary homonym of Dendromyrmex nidulans var. nigripes Wheeler, 1916: 13 (current combination *Camponotus nidulans nigripes*), junior synonym of *Camponotus nidulans* (Smith, 1860c: 69).

##### Type locality.

Malaysia: Perak, Belum.

##### Type material.

Holotype worker.

##### Type depository.

NHMB.

##### Etymology.

Arbitrary combination of letters, treated as a noun in apposition.

#### ﻿D5. Camponotus planus brina

Taxon classificationAnimaliaHymenopteraFormicidae

Fisher nom. nov.

B1E7A43F-090C-559B-975F-5619C2928ABB

##### Note.

Replacement name for Camponotus (Myrmorhachis) planus var. indefessus Wheeler, 1919: 294, junior secondary homonym of *Formica indefessa* Sykes, 1835: 104 (current combination *Camponotus indefessus*), junior synonym of *Camponotus compressus* (Fabricius, 1787: 307).

##### Type locality.

Ecuador: Galapagos Is, Indefatigable I. (= Santa Cruz Is.).

##### Type material.

Syntype workers.

##### Type depository.

MCZC.

##### Etymology.

Arbitrary combination of letters, treated as a noun in apposition.

#### ﻿D6. Carebara dazia

Taxon classificationAnimaliaHymenopteraFormicidae

Fisher nom. nov.

7E1B7332-32E0-506B-BFDB-875E911F737E

##### Note.

Replacement name for *Carebara laeviceps* Liu & Zhong, 2024: 17, junior secondary homonym of Oligomyrmex (Hendecatella) capreolus subsp. laeviceps Wheeler, 1928: 24 (current combination *Carebara capreola laeviceps*).

##### Type locality.

China: Sichuan Province, Kaijiang County, Dazhou City.

##### Type material.

Holotype worker.

##### Type depository.

SWFU.

##### Etymology.

Arbitrary combination of letters, treated as a noun in apposition.

#### ﻿D7. Carebara xynera

Taxon classificationAnimaliaHymenopteraFormicidae

Fisher nom. nov.

200279E9-019A-54A8-A6DF-9E1115F0F66B

##### Note.

Replacement name for *Aneleus politus* Santschi, 1914a: 79 (current combination *Carebara polita*), junior secondary homonym of *Myrmica polita* Smith, 1860b: 108 (current combination *Carebara polita*), junior synonym of *Carebara diversa* (Jerdon, 1851: 109).

##### Type locality.

Kenya (“Afrique orientale anglaise”): Blue Post Hotel, Kikuyu territory.

##### Type material.

Syntype workers.

##### Type depository.

NHMB.

##### Etymology.

Arbitrary combination of letters, treated as a noun in apposition.

#### ﻿D8. Colobopsis vitrea yevira

Taxon classificationAnimaliaHymenopteraFormicidae

Fisher nom. nov.

A16CCF88-09BE-5C85-8E5E-F5631C2C62D3

##### Note.

Replacement name for Camponotus (Colobopsis) vitreus r. carinata Stitz, 1938: 116 (current combination *Colobopsis vitrea carinata*), junior secondary homonym of *Colobopsis carinata* Mayr, 1870: 943, homonym replaced by *Colobopsis polynesica* (Emery, 1896: 374).

##### Type locality.

Papua New Guinea (“German New Guinea”): Aprilfluss.

##### Type material.

Syntype queens.

##### Type depository.

MNHU.

##### Etymology.

Arbitrary combination of letters, treated as a noun in apposition.

#### ﻿D9. Formica draven

Taxon classificationAnimaliaHymenopteraFormicidae

Fisher nom. nov.

56C84074-9D3A-58DC-82B7-D7F8C075F84C

##### Note.

Replacement name for Myrmecocystus melliger subsp. californicus Creighton, 1950: 445 (current combination *Formica californica*), junior secondary homonym of Formica microgyna subsp. californica Wheeler, 1917: 543, junior synonym of *Formica adamsi whymperi* Wheeler, 1917: 544.

##### Type locality.

USA: California, Weed.

##### Type material.

Holotype worker.

##### Type depository.

LACM.

##### Etymology.

Arbitrary combination of letters, treated as a noun in apposition.

#### ﻿D10. Messor draxil

Taxon classificationAnimaliaHymenopteraFormicidae

Fisher nom. nov.

99299ED5-CD44-5208-94E2-A5B4690D248B

##### Note.

Replacement name for *Messor sculpturatus* Carpenter, 1930: 32, junior secondary homonym of *Cratomyrmex sculpturatus* Stitz, 1916: 377 (current combination *Messor regalis sculpturatus*) junior synonym of *Messor regalis* (Emery, 1892b: 572).

##### Type locality.

USA: Colorado (Florissant Fossil Beds) Eocene.

##### Type material.

Fossil. fossil imprints (compression fossils). Holotype ♀ No. 2920, paratypes Nos. 2921 (MCZC), 10,032 (Peabody Museum), 7850 (Princeton University), 17,017a (University of Colorado), 78,804 (USNM), 11 (Carnegie Museum).

##### Type depository.

MCZC.

##### Etymology.

Arbitrary combination of letters, treated as a noun in apposition.

#### ﻿D11. Pheidole singaporensis zolara

Taxon classificationAnimaliaHymenopteraFormicidae

Fisher nom. nov.

50E3FBEA-3314-5A64-9B84-34C1C9E13A7A

##### Note.

Replacement name for Aphaenogaster (Ischnomyrmex) longipes var. continentis Forel, 1911a: 24 (current combination *Pheidole singaporensis continentis*), junior secondary homonym of Pheidole tasmaniensis var. continentis Forel, 1902c: 437.

##### Type locality.

Myanmar (“Burma”).

##### Type material.

Syntype minor workers.

##### Type depository.

MHNG.

##### Etymology.

Arbitrary combination of letters, treated as a noun in apposition.

#### ﻿D12. Pseudolasius zynia

Taxon classificationAnimaliaHymenopteraFormicidae

Fisher nom. nov.

6733C03E-E3B4-5FE1-A977-8A1E303D893A

##### Note.

Replacement name for *Rhizomyrma emeryi* Forel, 1915: 347 (current combination *Pseudolasius emeryi*), junior secondary homonym of *Pseudolasius emeryi* Forel, 1911c: 286.

##### Type locality.

Papua New Guinea: Hansemann Mts.

##### Type material.

Holotype worker.

##### Type depository.

MHNG.

##### Etymology.

Arbitrary combination of letters, treated as a noun in apposition.

#### ﻿D13. Tetramorium drunex

Taxon classificationAnimaliaHymenopteraFormicidae

Fisher nom. nov.

627D1900-52A1-55C5-8DA4-3B9AE023C442

##### Note.

Replacement name for *Tetramorium flavum* Chang & He, 2001: 2, junior secondary homonym of Xiphomyrmex gambogecus var. flavus Donisthorpe, 1941a: 58 (current combination *Tetramorium gambogecum flavum*), junior synonym of *Tetramorium gambogecum* (Donisthorpe, 1941a: 57).

##### Type locality.

China: Pengyang, Ningxia.

##### Type material.

Holotype worker.

##### Type depository.

NUYC.

##### Etymology.

Arbitrary combination of letters, treated as a noun in apposition.

#### ﻿D14. Tetramorium fenix

Taxon classificationAnimaliaHymenopteraFormicidae

Fisher nom. nov.

D715584C-B452-5DB4-BDC0-4AF12C400E87

##### Note.

Replacement name for *Tetramorium intermedium*Hita Garcia et al., 2010: 48, junior secondary homonym of Rhoptromyrmex rothneyi var. intermedia Forel, 1913b: 80 (current combination *Tetramorium rothneyi intermedium*), junior synonym of *Tetramorium wroughtonii* (Forel, 1902a: 231).

##### Type locality.

Ghana: Aiyeola Forest Reserve, Kade.

##### Type material.

Holotype worker.

##### Type depository.

NHMUK.

##### Etymology.

Arbitrary combination of letters, treated as a noun in apposition.

#### ﻿D15. Tetramorium flinex

Taxon classificationAnimaliaHymenopteraFormicidae

Fisher nom. nov.

A02BC478-E3DA-5BA8-A9B3-7DFD8AD28709

##### Note.

Replacement name for *Teleutomyrmex kutteri* Tinaut, 1990: 202 (current combination *Tetramorium kutteri*), junior secondary homonym of Tetramorium semilaeve var. kutteri Santschi, 1927a: 57, junior synonym of *Tetramorium indocile* Santschi, 1927a: 53.

##### Type locality.

Spain: Granada, Sierra Nevada, “Prados de Otero”.

##### Type material.

Holotype queen.

##### Type depository.

ATPC.

##### Etymology.

Arbitrary combination of letters, treated as a noun in apposition.

#### ﻿D16. Temnothorax fynor

Taxon classificationAnimaliaHymenopteraFormicidae

Fisher nom. nov.

65ED528F-23A3-5780-9FE7-75FAEA17DD13

##### Note.

Replacement name for *Temnothorax similis*Csősz et al., 2015: 23, junior secondary homonym of Leptothorax (Macromischa) similis Baroni Urbani, 1978: 501 (current combination *Temnothorax similis*), junior synonym of *Temnothorax laetus* (Wheeler, 1937: 456).

##### Type locality.

Turkey: Nur Daglari.

##### Type material.

Holotype worker.

##### Type depository.

HNHM.

##### Etymology.

Arbitrary combination of letters, treated as a noun in apposition.

#### ﻿D17. Tetraponera vantix

Taxon classificationAnimaliaHymenopteraFormicidae

Fisher nom. nov.

EF819181-974D-5987-8BD3-CA2646D85EE2

##### Note.

Replacement name for *Tetraponera insularis* Ward, 2022: 49, junior secondary homonym of Sima nigra var. insularis Emery, 1901b: 113 (current combination *Tetraponera nigra insularis*), junior synonym of *Tetraponera nigra* (Jerdon, 1851: 112).

##### Type locality.

Madagascar: Mahajanga, Forêt Anabohazo.

##### Type material.

Holotype worker.

##### Type depository.

CASC.

##### Etymology.

Arbitrary combination of letters, treated as a noun in apposition.

### ﻿E. Junior homonyms

In accordance with ICZN Articles 57.3 and 59.1, the following junior secondary homonyms are not replaced. In these cases, either (i) the senior homonym is a junior synonym and no longer valid, thus eliminating the conflict, or (ii) the taxonomic status is unresolved and a replacement would be premature. Replacement names are proposed only when required to resolve homonymy between valid, congeneric names in current use.

**E1.** No replacement name for *Monomorium bidentatum* Mayr, 1887: 616 (current combination *Monomorium bidentatum*), junior secondary homonym of *Myrmica bidentata* Smith, 1858: 124 (current combination *Monomorium bidentata*). The combination of *Monomorium bidentata* Smith, 1858 in *Monomorium* is unconvincing but even if correct, then a junior synonym of *Monomorium bidentatum* Mayr, 1887 exists (*Monomorium bidentatum piceonigrum* Borgmeier, 1948: 468).

**Type locality.** Chile: Valdivia.

**Type material.** Syntype workers, syntype queens (numbers not stated).

**Type depository.**NHMW.

**E2.** See Suppl. material 1 of 155 species and subspecies where unresolved junior homonyms are not replaced because the status of the junior homonym is unidentifiable (24 species, 2 subspecies) or because the junior homonym is treated as a junior synonym of another valid taxon (55 species, 74 subspecies).

### ﻿F. Supplementary taxonomic notes

**F1.** Reinstatement of *Tetramorium schultzei* Forel, 1910a, stat. nov. Examination of the holotype of Tetramorium caespitum subsp. schultzei Forel, 1910a: 19 (worker), together with a review of its taxonomic history, indicates that this taxon is not conspecific with *T. mossamedense*. Consequently, *T. schultzei* is here reinstated from synonymy and elevated to species rank (stat. nov.).

**Type locality.** Botswana: Kalahari, Kgokong-Kang, no. 966 (*L. Schultze*).

**Type material.** Holotype worker (AntWeb unique specimen identifier: FoCol2071 https://www.antweb.org/specimen/FoCol2071).

**Type depository.**MNHU.

The type was presumed lost by Bolton, 1980: 314; but B. Bolton (pers. comm. 17 April 2025) has noted that the *schultzei* holotype is not conspecific with *mossamedense*. *Tetramorium schultzei* is here reinstated and raised to species rank.

﻿*Tetramorium schultzei* Forel, 1910a, stat. nov.

Subspecies of *caespitum*: Santschi 1914b: 367; Arnold 1917: 331; Wheeler, W.M. 1922a: 894; Emery 1924: 278.

Junior synonym of *mossamedense*: Bolton 1980: 314 (mistake); Bolton 1995: 414 (mistake).

**F2.** New genus combinations from male-based reevaluations. Based on a detailed morphological reexamination of male specimens that remained in *Pachycondyla* following the treatment of Schmidt and Shattuck (2014), the following new genus combinations are proposed.

﻿**F2.1 *Bothroponera jonesii* (Forel, 1891), comb. nov.**

*Lobopelta jonesii* Forel, 1891 219 (m.) Madagascar.

**Type locality.** Madagascar: Forêt d’Andrangoloaka (F. Sikora).

**Type material.** Holotype male (AntWeb unique specimen identifier: CASENT0101709).

**Type depository.**MHNG.

﻿Combination in *Leptogenys* by Emery (1911: 102).

Combination in *Pachycondyla* by Bolton (1975: 298).

Incertae sedis in *Pachycondyla* by Schmidt and Shattuck (2014: 155).

﻿**F2.2 *Diacamma unicolor* (Smith, 1860a), comb. nov.**

*Ponera unicolor* Smith, 1860a 73 (m.) Indonesia (Sulawesi).

**Type locality.** Indonesia: Indonesia: Sulawesi (“Celebes”, Makassar, “Mak.” (A.R. Wallace).

**Type material.** Holotype male (apex of gaster missing) (AntWeb unique specimen identifier: CASENT0901368).

**Type depository.**OXUM.

Donisthorpe (1932: 458) cites 1 male in OXUM (see CASENT0901368). 2) There is a second male in OXUM, from Tondano (Sulawesi), apparently of the same species and complete (Bolton unpublished notes, 1978).

Combination in Pachycondyla (Bothroponera) by Donisthorpe (1932: 458).

Combination in *Bothroponera* by Joma and Mackay (2013: 2).

Incertae sedis in *Pachycondyla* by Schmidt and Shattuck (2014: 155).

﻿**F2.3 *Pseudoneoponera vidua* (Smith, 1857), comb. nov.**

*Ponera vidua* Smith, 1857 68 (m.) Borneo (East Malaysia: Sarawak).

**Type locality.** Malaysia: Borneo, Sarawak, “SAR.” (A.R. Wallace).

**Type material.** Holotype male (gaster missing) (AntWeb unique specimen identifier: CASENT0901367).

**Type depository.**OXUM.

Donisthorpe (1932: 448) cites 1 male in OXUM (see CASENT0901367).

Combination in *Pachycondyla* by Donisthorpe (1932: 448).

Incertae sedis in *Pachycondyla* by Schmidt and Shattuck (2014: 155).

﻿**F2.4 *Diacamma solitaria* (Smith, 1860b), comb. nov.**

*Ponera solitaria* Smith, 1860b 103 (m.) Indonesia (Bacan).

**Type locality.** Indonesia: Bachian (= Bacan I.), “Bac.” (A.R. Wallace).

**Type material.** Holotype male (head detached, glued separately onto the stage card) (AntWeb unique specimen identifier: CASENT0901366).

**Type depository.**OXUM.

**Type notes.** Donisthorpe, 1932: 461, cites 1 male in OXUM (see CASENT0901366).

Combination in Pachycondyla (Bothroponera) by Donisthorpe (1932: 461).

Combination in *Bothroponera* by Wilson (1958: 362); Joma and Mackay (2013: 2).

Combination in *Pachycondyla* by Brown (1995: 309).

Incertae sedis in *Pachycondyla* by Schmidt and Shattuck (2014: 155).

## Supplementary Material

XML Treatment for Estevia

XML Treatment for Estevia megagastrosa

XML Treatment for Acromyrmex atrina

XML Treatment for Camponotus dorex

XML Treatment for Camponotus durnyx

XML Treatment for Camponotites flarex

XML Treatment for Camponotus robecchii clyne

XML Treatment for Camponotus substitutus cylrix

XML Treatment for Colobopsis breva

XML Treatment for Crematogaster scutellaris gynia

XML Treatment for Dolichoderus thoracicus jexina

XML Treatment for Ectomomyrmex pylixy

XML Treatment for Ectomomyrmex pylor

XML Treatment for Euponera tynara

XML Treatment for Formica lavateri liora

XML Treatment for Formica mytara

XML Treatment for Hypoponera rylin

XML Treatment for Hypoponera veltan

XML Treatment for Lepisiota vynia

XML Treatment for Temnothorax flavispinus novira

XML Treatment for Messor minor nubina

XML Treatment for Messor nuvia

XML Treatment for Pheidole arnoldi qindra

XML Treatment for Pseudoneoponera sublaevis quorra

XML Treatment for Strumigenys wylixa

XML Treatment for Camponotus chucki

XML Treatment for Camponotus foleyi cybira

XML Treatment for Camponotus maculatus brexyl

XML Treatment for Camponotus nixra

XML Treatment for Camponotus planus brina

XML Treatment for Carebara dazia

XML Treatment for Carebara xynera

XML Treatment for Colobopsis vitrea yevira

XML Treatment for Formica draven

XML Treatment for Messor draxil

XML Treatment for Pheidole singaporensis zolara

XML Treatment for Pseudolasius zynia

XML Treatment for Tetramorium drunex

XML Treatment for Tetramorium fenix

XML Treatment for Tetramorium flinex

XML Treatment for Temnothorax fynor

XML Treatment for Tetraponera vantix

## ﻿Additional information

### Use of AI

AI tools build into MS Word were used for language editing and formatting assistance under the author’s supervision.

### Author ORCIDs

Brian L. Fisher ￼ https://orcid.org/0000-0002-4653-3270

### Data availability

All of the data that support the findings of this study are available in the main text or Supplementary Information.

## Supplementary materials

Supplementary material 1 The 155 species-group names where unresolved junior homonyms are not replaced because the status of the junior homonym is unidentifiable (24 species, 2 subspecies) or a synonym (55 species, 74 subspecies). Data type docx https://binary.pensoft.net/file/1441116This dataset is made available under the Open Database License (http://opendatacommons.org/licenses/odbl/1.0/). The Open Database License (ODbL) is a license agreement intended to allow users to freely share, modify, and use this Dataset while maintaining this same freedom for others, provided that the original source and author(s) are credited.Author: Brian L. Fisher
