# Supplementary material for: ﻿Replacement names for junior homonyms in ants (Hymenoptera, Formicidae)
Source: Zookeys. 2025 Oct 16;1256:81–113. doi: 10.3897/zookeys.1256.162607 (PMC12550506; doi:10.3897/zookeys.1256.162607)
Supplement: Supplementary material 1 — The 155 species-group names where unresolved junior homonyms are not replaced because the status of the junior homonym is unidentifiable (24 species, 2 subspecies) or a synonym (55 species, 74 subspecies). [file zookeys-1256-081_article-162607__-s001.docx]

**Replacement names for junior homonyms in Ants (Hymenoptera, Formicidae)**

Brian L. Fisher

Department of Entomology, California Academy of Sciences, San Francisco, California, USA

Orcid: 0000-0002-4653-327

**Supplementary Material**

**Supplementary Table 1.** The 155 species-group names where unresolved junior homonyms are not replaced because the status of the junior homonym is unidentifiable (24 species, 2 subspecies) or a synonym (55 species, 74 subspecies). If future taxonomic work recognizes them as valid species-group taxa, replacement names will be necessary. References included below are available on AntCat.org.

| **Original / current combination** | **Author** | **Rank** | **Status** | **Unresolved** **Homonym** |
| --- | --- | --- | --- | --- |
| [*Acromyrmex octospinosa var. pallida*](https://antcat.org/protonyms/156091) [*Acromyrmex octospinosus pallida*](https://antcat.org/catalog/430361) | Crawley, 1921 | subspecies | synonym | Unresolved junior secondary homonym of [*Acromyrmex pallida*](https://antcat.org/catalog/430360) [Smith, 1858a](https://antcat.org/references/128685) ([Bolton, 1995b](https://antcat.org/references/122860): 56). |
| [*Anochetus* (*Stenomyrmex*) *emarginatus r. rugosus*](https://antcat.org/protonyms/156650) [*Anochetus emarginatus rugosus*](https://antcat.org/catalog/430920) | Emery, 1890b | subspecies | synonym | Unresolved junior secondary homonym of *[Anochetus rugosus](https://antcat.org/catalog/430919)* [Smith, 1857a](https://antcat.org/references/128683) ([Bolton, 1995b](https://antcat.org/references/122860): 65). |
| [*Anochetus rossi*](https://antcat.org/protonyms/156644) [*Anochetus rossi*](https://antcat.org/catalog/430914) | Donisthorpe, 1949c | species | synonym | Unresolved junior primary homonym of *[Anochetus rossi](https://antcat.org/catalog/430913)* [Donisthorpe, 1947e](https://antcat.org/references/124354) ([Bolton, 1995b](https://antcat.org/references/122860): 65). |
| [*Antillaemyrmex floridanus*](https://antcat.org/protonyms/175420) [*Temnothorax floridanus*](https://antcat.org/catalog/449690) | Wheeler, 1931b | species | synonym | Unresolved junior secondary homonym of [*Temnothorax pergandei floridanus*](https://antcat.org/catalog/511359) [Emery, 1895d](https://antcat.org/references/124591) ([Bolton, 1995b](https://antcat.org/references/122860): 238). |
| [*Aphaenogaster* (*Planimyrma*) *dromedarius var. nigra*](https://antcat.org/protonyms/156925) [*Aphaenogaster dromedaria nigra*](https://antcat.org/catalog/431195) | Donisthorpe, 1938b | subspecies | synonym | Unresolved junior primary homonym of *[Messor barbarus nigra](https://antcat.org/catalog/440141)* [André, 1883b](https://antcat.org/references/122254) ([Bolton, 1995b](https://antcat.org/references/122860): 71). |
| [*Aphaenogaster semipolita subsp. ionia*](https://antcat.org/protonyms/156870) [*Aphaenogaster ionia*](https://antcat.org/catalog/512789) | Baroni Urbani, 1968e | species | synonym | Unresolved junior primary homonym of [*Aphaenogaster ionia*](https://antcat.org/catalog/431139) [Santschi, 1933d](https://antcat.org/references/128413) ([Bolton, 1995b](https://antcat.org/references/122860): 70). |
| [*Aphaenogaster striola var. laevior*](https://antcat.org/protonyms/156881) [*Aphaenogaster gibbosa laevior*](https://antcat.org/catalog/512790) | Forel, 1892g | subspecies | synonym | Unresolved junior primary homonym of [*Aphaenogaster laevior*](https://antcat.org/catalog/431150) [Emery, 1887g](https://antcat.org/references/142688) ([Bolton, 1995b](https://antcat.org/references/122860): 70). |
| [*Belonopelta darwinii var. indica*](https://antcat.org/protonyms/168662) [*Parvaponera darwinii indica*](https://antcat.org/catalog/442932) | Forel, 1900f | subspecies | synonym | Unresolved junior primary homonym of *[Parvaponera darwinii indica](https://antcat.org/catalog/442931)* [Emery, 1899e](https://antcat.org/references/124633). |
| [*Camponotus* (*Myrmamblys*) *aurofasciatus*](https://antcat.org/protonyms/157785) [*Camponotus aurofasciatus*](https://antcat.org/catalog/432055) | Wheeler, 1915h | species | synonym | Unresolved junior primary homonym of [*Camponotus aurofasciatus*](https://antcat.org/catalog/432054) [Santschi, 1915c](https://antcat.org/references/128292) ([Bolton, 1995b](https://antcat.org/references/122860): 87). |
| [*Camponotus* (*Myrmosericus*) *feae var. erythropus*](https://antcat.org/protonyms/158262) [*Camponotus feae erythropus*](https://antcat.org/catalog/432532) | Stitz, 1917 | subspecies | synonym | Unresolved junior primary homonym of [*Camponotus erythropus*](https://antcat.org/catalog/432531) [Pergande, 1893](https://antcat.org/references/127765) ([Bolton, 1995b](https://antcat.org/references/122860): 98). |
| [*Camponotus christi var. ferruginea*](https://antcat.org/protonyms/158320) [*Camponotus christi ferrugineus*](https://antcat.org/catalog/432590) | Emery, 1899e | subspecies | synonym | Unresolved junior secondary homonym of [*Camponotus ferrugineus*](https://antcat.org/catalog/432589) [Fabricius, 1798](https://antcat.org/references/124869) ([Bolton, 1995b](https://antcat.org/references/122860): 99). |
| [*Camponotus herculeanus var. montanus*](https://antcat.org/protonyms/158927) [*Camponotus herculeanus montanus*](https://antcat.org/catalog/433197) | Ruzsky, 1904a | subspecies | synonym | Unresolved junior primary homonym of [*Camponotus alboannulatus montanus*](https://antcat.org/catalog/433196) [Emery, 1894d](https://antcat.org/references/124576) ([Bolton, 1995b](https://antcat.org/references/122860): 112). |
| [*Camponotus karawajewi*](https://antcat.org/protonyms/158661) [*Camponotus karawajewi*](https://antcat.org/catalog/432931) | Radchenko, 1996e | species | synonym | Unresolved junior primary homonym of *[Colobopsis karawaiewi](https://antcat.org/catalog/507319)* [Menozzi, 1926e](https://antcat.org/references/127332). |
| [*Camponotus lateralis var. rectus*](https://antcat.org/protonyms/159290) [*Camponotus rectus*](https://antcat.org/catalog/513351) | Forel, 1892j | species | synonym | Unresolved junior primary homonym of [*Camponotus lubbocki rectus*](https://antcat.org/catalog/433559) [Forel, 1891c](https://antcat.org/references/125030) ([Bolton, 1995b](https://antcat.org/references/122860): 120). |
| [*Camponotus maculatus subsp. ruzskyi*](https://antcat.org/protonyms/159369) [*Camponotus maculatus ruzskyi*](https://antcat.org/catalog/433639) | Vashkevich, 1924a | subspecies | synonym | Unresolved junior primary homonym of [*Camponotus ruzskyi*](https://antcat.org/catalog/433638) [Emery, 1898c](https://antcat.org/references/124628) ([Bolton, 1995b](https://antcat.org/references/122860): 121). |
| [*Camponotus sylvaticus var. dichrous*](https://antcat.org/protonyms/158186) [*Camponotus dichrous*](https://antcat.org/catalog/432456) | André, 1882a | species | synonym | Note: if synonymy confirmed then [*Camponotus dichrous*](https://antcat.org/catalog/432456) is the first available replacement name for [*Camponotus thoracicus*](https://antcat.org/catalog/433879) [Fabricius, 1804](https://antcat.org/references/124870), unresolved junior primary homonym of [*Formica thoracica*](https://antcat.org/catalog/437838) [Olivier, 1792](https://antcat.org/references/127622). |
| [*Cardiocondyla brevispinosa*](https://antcat.org/protonyms/159793) [*Cardiocondyla brevispinosa*](https://antcat.org/catalog/434063) | Weber, 1952a | species | synonym | Unresolved junior secondary homonym of *[Cardiocondyla brevispinosa](https://antcat.org/catalog/434062)* [Donisthorpe, 1948d](https://antcat.org/references/124360) ([Bolton, 1982](https://antcat.org/references/122828): 316). |
| [*Crematogaster* (*Orthocrema*) *curvispinosa var. obscura*](https://antcat.org/protonyms/161540) [*Crematogaster curvispinosa obscura*](https://antcat.org/catalog/435810) | Santschi, 1929d | subspecies | synonym | Unresolved junior primary homonym of [*Crematogaster obscura*](https://antcat.org/catalog/435808) [Smith, 1857a](https://antcat.org/references/128683) ([Bolton, 1995b](https://antcat.org/references/122860): 159). |
| [*Croesomyrmex iris var. tristis*](https://antcat.org/protonyms/175771) [*Temnothorax iris tristis*](https://antcat.org/catalog/450041) | Wheeler, 1931b | subspecies | synonym | Unresolved junior secondary homonym of [*Temnothorax tristis*](https://antcat.org/catalog/450040) [Bondroit, 1918](https://antcat.org/references/122872) ([Bolton, 1995b](https://antcat.org/references/122860): 246). |
| [*Dendromyrmex apicalis var. opaciceps*](https://antcat.org/protonyms/159075) [*Camponotus apicalis opaciceps*](https://antcat.org/catalog/433345) | Wheeler, 1923a | subspecies | synonym | Unresolved junior secondary homonym of [*Camponotus opaciceps*](https://antcat.org/catalog/433344) [Roger, 1863a](https://antcat.org/references/128093). |
| [*Dendromyrmex fabricii subsp. niger*](https://antcat.org/protonyms/158979) [*Camponotus fabricii niger*](https://antcat.org/catalog/433249) | Weber, 1943b | subspecies | synonym | Unresolved junior secondary homonym of [*Camponotus sicheli nigra*](https://antcat.org/catalog/433248) [Emery, 1925d](https://antcat.org/references/124775). |
| [*Dendromyrmex fabricii var. pictus*](https://antcat.org/protonyms/159173) [*Camponotus fabricii pictus*](https://antcat.org/catalog/433443) | Wheeler, 1923a | subspecies | synonym | Unresolved junior secondary homonym of [*Camponotus ligniperda pictus*](https://antcat.org/catalog/433442) [Forel, 1886h](https://antcat.org/references/125017). |
| [*Dendromyrmex madeirensis var. romani*](https://antcat.org/protonyms/159321) [*Camponotus madeirensis romani*](https://antcat.org/catalog/433591) | Wheeler, 1923a | subspecies | synonym | Unresolved junior secondary homonym of [*Camponotus abdominalis romani*](https://antcat.org/catalog/433590) [Wheeler, 1923a](https://antcat.org/references/130163). |
| [*Dendromyrmex mamoreensis st. vestita*](https://antcat.org/protonyms/159701) [*Camponotus mamoreensis vestitus*](https://antcat.org/catalog/433971) | Santschi, 1921g | subspecies | synonym | Unresolved junior secondary homonym of [*Camponotus vestitus*](https://antcat.org/catalog/433970) [Smith, 1858a](https://antcat.org/references/128685). |
| [*Dendromyrmex wheeleri*](https://antcat.org/protonyms/159740) [*Camponotus wheeleri*](https://antcat.org/catalog/434010) | Donisthorpe, 1937a | species | synonym | Unresolved junior secondary homonym of [*Camponotus wheeleri*](https://antcat.org/catalog/434009) [Mann, 1916](https://antcat.org/references/127070). |
| [*Descolemyrma ogloblini*](https://antcat.org/protonyms/166663) [*Mycocepurus ogloblini*](https://antcat.org/catalog/440933) | Kusnezov, 1951g | species | synonym | Unresolved junior secondary homonym of *[Mycocepurus ogloblini](https://antcat.org/catalog/440932)* [Santschi, 1933f](https://antcat.org/references/128415) ([Bolton, 1995b](https://antcat.org/references/122860): 269). |
| [*Dolichoderus* (*Hypoclinea*) *sulcaticeps var. nigriventris*](https://antcat.org/protonyms/162365) [*Dolichoderus sulcaticeps nigriventris*](https://antcat.org/catalog/436635) | Santschi, 1920h | subspecies | synonym | Unresolved junior primary homonym of [*Dolichoderus lutosus nigriventris*](https://antcat.org/catalog/436634) [Forel, 1893j](https://antcat.org/references/125057) ([Bolton, 1995b](https://antcat.org/references/122860): 175). |
| [*Eciton* (*Acamatus*) *californicum var. obscura*](https://antcat.org/protonyms/167766) [*Neivamyrmex californicus obscurus*](https://antcat.org/catalog/442036) | Forel, 1914d | subspecies | synonym | Unresolved junior primary homonym of *[Neivamyrmex spinolae obscurus](https://antcat.org/catalog/442035)* [Forel, 1911g](https://antcat.org/references/125216) ([Bolton, 1995b](https://antcat.org/references/122860): 290). |
| [*Eciton* (*Aenictus*) *fergusoni var. sundaica*](https://antcat.org/protonyms/156435) [*Aenictus sundaica*](https://antcat.org/catalog/508216) | Karavaiev, 1927f | species | synonym | Unresolved junior secondary homonym of *[Aenictus clavatus sundaicus](https://antcat.org/catalog/430704)* [Forel, 1909i](https://antcat.org/references/125197) ([Bolton, 1995b](https://antcat.org/references/122860): 60). |
| [*Ectatomma* (*Gnamptogenys*) *continuum var. panamensis*](https://antcat.org/protonyms/163803) [*Gnamptogenys continua panamensis*](https://antcat.org/catalog/438073) | Santschi, 1931d | subspecies | synonym | Unresolved junior primary homonym of *[Heteroponera panamensis](https://antcat.org/catalog/438216)* [Forel, 1899b](https://antcat.org/references/125089) ([Bolton, 1995b](https://antcat.org/references/122860): 210). |
| [*Ectatomma* (*Rhytidoponera*) *impressum var. splendidum*](https://antcat.org/protonyms/173153) [*Rhytidoponera impressa splendidum*](https://antcat.org/catalog/447423) | Forel, 1910b | subspecies | synonym | Unresolved junior primary homonym of *[Gnamptogenys regularis splendidum](https://antcat.org/catalog/511134)* [Pergande, 1896](https://antcat.org/references/127767) ([Bolton, 1995b](https://antcat.org/references/122860): 380). |
| [*Ectatomma* (*Stictoponera*) *bicolor var. minor*](https://antcat.org/protonyms/163787) [*Stictoponera menadensis minor*](https://antcat.org/catalog/512991) | Forel, 1900f | subspecies | synonym | Unresolved junior primary homonym of *[Acanthoponera minor](https://antcat.org/catalog/430235)* [Forel, 1899b](https://antcat.org/references/125089) ([Bolton, 1995b](https://antcat.org/references/122860): 209). |
| [*Emeryella schmitti subsp. minor*](https://antcat.org/protonyms/163788) [*Gnamptogenys schmitti minor*](https://antcat.org/catalog/438058) | Wheeler, 1936c | subspecies | synonym | Unresolved junior secondary homonym of *[Gnamptogenys bicolor minor](https://antcat.org/catalog/438057)* [Forel, 1900f](https://antcat.org/references/125101) ([Bolton, 1995b](https://antcat.org/references/122860): 209). |
| [*Eneria excisa*](https://antcat.org/protonyms/174287) [*Strumigenys excisa*](https://antcat.org/catalog/448557) | Donisthorpe, 1948d | species | synonym | Unresolved junior secondary homonym of [*Strumigenys excisa*](https://antcat.org/catalog/448556) [Weber, 1934a](https://antcat.org/references/129709). |
| [*Epimyrma foreli*](https://antcat.org/protonyms/167604) [*Temnothorax foreli*](https://antcat.org/catalog/461983) | Menozzi, 1921 | species | synonym | Unresolved junior secondary homonym of [*Temnothorax foreli*](https://antcat.org/catalog/449691) [Santschi, 1907](https://antcat.org/references/128244). |
| [*Epitritus clypeatus*](https://antcat.org/protonyms/174166) [*Strumigenys clypeata*](https://antcat.org/catalog/448436) | Szabó, 1909 | species | synonym | Unresolved junior secondary homonym of [*Strumigenys clypeata*](https://antcat.org/catalog/448435) [Roger, 1863a](https://antcat.org/references/128093). |
| [*Formica* (*Formica*) *rufa var. nuda*](https://antcat.org/protonyms/163379) [*Formica rufa nuda*](https://antcat.org/catalog/437649) | Karavaiev, 1930b | subspecies | synonym | Unresolved junior primary homonym of [*Formica pratensis nuda*](https://antcat.org/catalog/437648) [Ruzsky, 1926](https://antcat.org/references/128169) ([Bolton, 1995b](https://antcat.org/references/122860): 200). |
| [*Formica* (*Raptiformica*) *sanguinea var. strennua*](https://antcat.org/protonyms/163535) [*Formica sanguinea strennua*](https://antcat.org/catalog/437805) | Santschi, 1925g | subspecies | synonym | Unresolved junior primary homonym of *[Lasiophanes strenua](https://antcat.org/catalog/438705)* [Haliday, 1836](https://antcat.org/references/125734) ([Bolton, 1995b](https://antcat.org/references/122860): 204). |
| [*Formica* (*Raptiformica*) *sanguinea var. tristis*](https://antcat.org/protonyms/163583) [*Formica sanguinea tristis*](https://antcat.org/catalog/437853) | Karavaiev, 1929d | subspecies | synonym | Unresolved junior primary homonym of [*Formica tristis*](https://antcat.org/catalog/437852) [Christ, 1791](https://antcat.org/references/123624) ([Bolton, 1995b](https://antcat.org/references/122860): 205). |
| [*Formica* (*Serviformica*) *cinerea var. brevisetosa*](https://antcat.org/protonyms/163090) [*Formica cinerea brevisetosa*](https://antcat.org/catalog/437360) | Karavaiev, 1927a | subspecies | synonym | Unresolved junior primary homonym of [*Formica rufa brevisetosa*](https://antcat.org/catalog/437359) [Ruzsky, 1926](https://antcat.org/references/128169). |
| [*Formica affinis*](https://antcat.org/protonyms/171107) [*Polyrhachis affinis*](https://antcat.org/catalog/445377) | Le Guillou, 1842 | species | synonym | Unresolved junior primary homonym of [*Formica affinis*](https://antcat.org/catalog/437311) [Leach, 1825](https://antcat.org/references/126852) ([Bolton, 1995b](https://antcat.org/references/122860): 343). |
| [*Formica americana*](https://antcat.org/protonyms/157694) [*Camponotus americanus*](https://antcat.org/catalog/431964) | Buckley, 1866 | species | unidentifiable | Unresolved junior secondary homonym of [*Camponotus americanus*](https://antcat.org/catalog/431963) [Mayr, 1862](https://antcat.org/references/127190) ([Bolton, 1995b](https://antcat.org/references/122860): 85). |
| [*Formica atra*](https://antcat.org/protonyms/157759) [*Camponotus atra*](https://antcat.org/catalog/432029) | Buckley, 1866 | species | unidentifiable | Unresolved junior primary homonym of [*Camponotus atra*](https://antcat.org/catalog/432028) [Zetterstedt, 1838](https://antcat.org/references/130596) ([Bolton, 1995b](https://antcat.org/references/122860): 86). |
| [*Formica atra*](https://antcat.org/protonyms/163071) [*Formica atra*](https://antcat.org/catalog/437341) | Schilling, 1839 | species | unidentifiable | Unresolved junior primary homonym of [*Camponotus atra*](https://antcat.org/catalog/432028) [Zetterstedt, 1838](https://antcat.org/references/130596) ([Bolton, 1995b](https://antcat.org/references/122860): 191). |
| [*Formica bicolor*](https://antcat.org/protonyms/157847) [*Camponotus bicolor*](https://antcat.org/catalog/432117) | Latreille, 1798 | species | synonym | Unresolved junior primary homonym of [*Cataglyphis bicolor*](https://antcat.org/catalog/434390) [Fabricius, 1793](https://antcat.org/references/124868) ([Emery, 1921a](https://antcat.org/references/124747): 26; [Bolton, 1995b](https://antcat.org/references/122860): 88). |
| [*Formica bicolor*](https://antcat.org/protonyms/163079) [*Formica bicolor*](https://antcat.org/catalog/437349) | Leach, 1825 | species | unidentifiable | Unresolved junior primary homonym of [*Cataglyphis bicolor*](https://antcat.org/catalog/434390) [Fabricius, 1793](https://antcat.org/references/124868) ([Emery, 1921a](https://antcat.org/references/124747): 26; [Bolton, 1995b](https://antcat.org/references/122860): 192). |
| [*Formica binodis*](https://antcat.org/protonyms/201229) [*Formica binodis*](https://antcat.org/catalog/509592) | Forskål, 1775 | species | unidentifiable | Unresolved junior primary homonym of [*Tetramorium binodis*](https://antcat.org/catalog/450173) [Linnaeus, 1763](https://antcat.org/references/126905). |
| [*Formica binodis*](https://antcat.org/protonyms/165749) [*Messor binodis*](https://antcat.org/catalog/440019) | Fabricius, 1775 | species | synonym | Unresolved junior primary homonym of [*Tetramorium binodis*](https://antcat.org/catalog/450173) [Linnaeus, 1763](https://antcat.org/references/126905) ([Bolton, 1995b](https://antcat.org/references/122860): 252). |
| [*Formica distinguenda*](https://antcat.org/protonyms/201220) [*Camponotus distinguendus*](https://antcat.org/catalog/509511) | Smith, 1871a | species | synonym | Unresolved junior primary homonym of [*Camponotus distinguendus*](https://antcat.org/catalog/432468) [Spinola, 1851a](https://antcat.org/references/128974). |
| [*Formica exsecta var. wheeleri*](https://antcat.org/protonyms/163603) [*Formica exsecta wheeleri*](https://antcat.org/catalog/437873) | Stitz, 1939 | subspecies | unidentifiable | Unresolved junior primary homonym of [*Formica wheeleri*](https://antcat.org/catalog/437872) [Creighton, 1935](https://antcat.org/references/123892). |
| [*Formica fragilis*](https://antcat.org/protonyms/163201) [*Formica fragilis*](https://antcat.org/catalog/437471) | Heer, 1867 | species | unidentifiable | Unresolved junior primary homonym of [*Leptomyrmex fragilis*](https://antcat.org/catalog/439506) [Smith, 1859a](https://antcat.org/references/128687) ([Bolton, 1995b](https://antcat.org/references/122860): 195). |
| [*Formica fusca*](https://antcat.org/protonyms/176066) [*Tetramorium fusca*](https://antcat.org/catalog/450336) | Leach, 1825 | species | synonym | Unresolved junior primary homonym of [*Formica fusca*](https://antcat.org/catalog/437477) [Linnaeus, 1758](https://antcat.org/references/126902) ([Bolton, 1995b](https://antcat.org/references/122860): 408). |
| [*Formica fusca var. blanda*](https://antcat.org/protonyms/163083) [*Formica fusca blanda*](https://antcat.org/catalog/437353) | Wheeler, 1913i | subspecies | synonym | Unresolved junior primary homonym of [*Camponotus blandus*](https://antcat.org/catalog/432134) [Smith, 1858a](https://antcat.org/references/128685) ([Bolton, 1995b](https://antcat.org/references/122860): 192). |
| [*Formica fusca var. pallipes*](https://antcat.org/protonyms/163416) [*Formica fusca pallipes*](https://antcat.org/catalog/437686) | Kuznetsov-Ugamsky, 1926c | subspecies | synonym | Unresolved junior primary homonym of [*Formica pallipes*](https://antcat.org/catalog/437685) [Fabricius, 1787](https://antcat.org/references/124867) ([Bolton, 1995b](https://antcat.org/references/122860): 200). |
| [*Formica fusca var. rubescens*](https://antcat.org/protonyms/163481) [*Formica fusca rubescens*](https://antcat.org/catalog/437751) | Forel, 1904g | subspecies | synonym | Unresolved junior primary homonym of [*Formica rubescens*](https://antcat.org/catalog/437750) [Leach, 1825](https://antcat.org/references/126852) ([Bolton, 1995b](https://antcat.org/references/122860): 202). |
| [*Formica fusca var. rufipes*](https://antcat.org/protonyms/163488) [*Formica fusca rufipes*](https://antcat.org/catalog/437758) | Stitz, 1930a | subspecies | synonym | Unresolved junior primary homonym of [*Camponotus rufipes*](https://antcat.org/catalog/433625) [Fabricius, 1775](https://antcat.org/references/124865) ([Bolton, 1995b](https://antcat.org/references/122860): 203). |
| [*Formica gibbosa*](https://antcat.org/protonyms/163223) [*Formica gibbosa*](https://antcat.org/catalog/437493) | Presl, 1822 | species | unidentifiable | Unresolved junior primary homonym of [*Aphaenogaster gibbosa*](https://antcat.org/catalog/431119) [Latreille, 1798](https://antcat.org/references/126792) ([Bolton, 1995b](https://antcat.org/references/122860): 196). |
| [*Formica gigas*](https://antcat.org/protonyms/158437) [*Camponotus gigas*](https://antcat.org/catalog/508874) | Leach, 1825 | species | synonym | Unresolved junior primary homonym of *[Dinomyrmex gigas](https://antcat.org/catalog/507606)* [Latreille, 1802a](https://antcat.org/references/126800) ([Bolton, 1995b](https://antcat.org/references/122860): 102). |
| [*Formica glabra*](https://antcat.org/protonyms/163226) [*Formica glabra*](https://antcat.org/catalog/437496) | White, 1884 | species | synonym | Unresolved junior primary homonym of [*Formica glabra*](https://antcat.org/catalog/437495) [Gmelin, 1790](https://antcat.org/references/125505) ([Bolton, 1995b](https://antcat.org/references/122860): 196). |
| [*Formica gracilis*](https://antcat.org/protonyms/175040) [*Tapinoma gracile*](https://antcat.org/catalog/449310) | Buckley, 1866 | species | synonym | Unresolved junior primary homonym of *[Pseudomyrmex gracilis](https://antcat.org/catalog/447053)* [Fabricius, 1804](https://antcat.org/references/124870) ([Bolton, 1995b](https://antcat.org/references/122860): 400). |
| [*Formica incisa*](https://antcat.org/protonyms/163261) [*Formica incisa*](https://antcat.org/catalog/437531) | Smith, 1858a | species | unidentifiable | Unresolved junior primary homonym of *[Lasius incisus](https://antcat.org/catalog/438791)* [Schenck, 1852](https://antcat.org/references/128471) ([Emery, 1921a](https://antcat.org/references/124747): 26; [Bolton, 1995b](https://antcat.org/references/122860): 197). |
| [*Formica lutea*](https://antcat.org/protonyms/163307) [*Formica lutea*](https://antcat.org/catalog/437577) | Duméril, 1860 | species | unidentifiable | Unresolved junior primary homonym of [*Camponotus luteus*](https://antcat.org/catalog/433065) [Smith, 1858a](https://antcat.org/references/128685) ([Bolton, 1995b](https://antcat.org/references/122860): 198). |
| [*Formica macrocephala*](https://antcat.org/protonyms/163310) [*Lasius macrocephalus*](https://antcat.org/catalog/508869) | Heer, 1849 | species | synonym | Unresolved junior primary homonym of *[Colobopsis macrocephala](https://antcat.org/catalog/507334)* [Erichson, 1842](https://antcat.org/references/124787) ([Bolton, 1995b](https://antcat.org/references/122860): 198). |
| [*Formica maculata*](https://antcat.org/protonyms/163313) [*Formica maculata*](https://antcat.org/catalog/437583) | Geoffroy, 1785 | species | unidentifiable | Unresolved junior primary homonym of [*Camponotus maculatus*](https://antcat.org/catalog/433086) [Fabricius, 1782](https://antcat.org/references/124871) ([Bolton, 1995b](https://antcat.org/references/122860): 198). |
| [*Formica maxillosa*](https://antcat.org/protonyms/163326) [*Formica maxillosa*](https://antcat.org/catalog/437596) | Fabricius, 1775 | species | unidentifiable | Unresolved junior primary homonym of [*Odontomachus maxillosus*](https://antcat.org/catalog/442522) [De Geer, 1773](https://antcat.org/references/124032) ([Bolton, 1995b](https://antcat.org/references/122860): 198). |
| [*Formica megacephala*](https://antcat.org/protonyms/165860) [*Messor megacephala*](https://antcat.org/catalog/440130) | Leach, 1825 | species | synonym | Unresolved junior primary homonym of [*Pheidole megacephala*](https://antcat.org/catalog/444098) [Fabricius, 1793](https://antcat.org/references/124868) ([Emery, 1921a](https://antcat.org/references/124747): 26; [Bolton, 1995b](https://antcat.org/references/122860): 255). |
| [*Formica nigra*](https://antcat.org/protonyms/163369) [*Formica nigra*](https://antcat.org/catalog/437639) | Presl, 1822 | species | unidentifiable | Unresolved junior primary homonym of *[Lasius niger](https://antcat.org/catalog/438835)* [Linnaeus, 1758](https://antcat.org/references/126902) ([Bolton, 1995b](https://antcat.org/references/122860): 199). |
| [*Formica nigra*](https://antcat.org/protonyms/202258) [*Formica nigra*](https://antcat.org/catalog/513312) | Forskål, 1775 | species | unidentifiable | Unresolved junior primary homonym of *[Lasius niger](https://antcat.org/catalog/438835)* [Linnaeus, 1758](https://antcat.org/references/126902). |
| [*Formica obsoleta*](https://antcat.org/protonyms/159045) [*Camponotus obsoleta*](https://antcat.org/catalog/433315) | Christ, 1791 | species | synonym | Unresolved junior primary homonym of [*Formica obsoleta*](https://antcat.org/catalog/437657) [Linnaeus, 1758](https://antcat.org/references/126902) ([Bolton, 1995b](https://antcat.org/references/122860): 114). |
| [*Formica picea*](https://antcat.org/protonyms/168045) [*Nylanderia picea*](https://antcat.org/catalog/442315) | Buckley, 1866 | species | synonym | Unresolved junior primary homonym of [*Camponotus piceus*](https://antcat.org/catalog/433436) [Leach, 1825](https://antcat.org/references/126852). |
| [*Formica picipes*](https://antcat.org/protonyms/163435) [*Formica picipes*](https://antcat.org/catalog/437705) | Reich, 1793 | species | unidentifiable | Unresolved junior primary homonym of [*Camponotus picipes*](https://antcat.org/catalog/433437) [Olivier, 1792](https://antcat.org/references/127622) ([Bolton, 1995b](https://antcat.org/references/122860): 201). |
| [*Formica pratensis var. ciliata*](https://antcat.org/protonyms/163109) [*Formica pratensis ciliata*](https://antcat.org/catalog/437379) | Ruzsky, 1926 | subspecies | synonym | Unresolved junior primary homonym of [*Formica ciliata*](https://antcat.org/catalog/437378) [Mayr, 1886d](https://antcat.org/references/127240) ([Bolton, 1995b](https://antcat.org/references/122860): 192). |
| [*Formica rufa var. montana*](https://antcat.org/protonyms/163347) [*Formica rufa montana*](https://antcat.org/catalog/437617) | Sadil, 1953b | subspecies | synonym | Unresolved junior primary homonym of [*Formica montana*](https://antcat.org/catalog/437615) [Wheeler, 1910a](https://antcat.org/references/130044) ([Bolton, 1995b](https://antcat.org/references/122860): 199). |
| [*Formica rufa var. rufotruncicola*](https://antcat.org/protonyms/163497) [*Formica rufa rufotruncicola*](https://antcat.org/catalog/437767) | Ruzsky, 1896 | subspecies | synonym | Unresolved junior primary homonym of [*Formica rufotruncicola*](https://antcat.org/catalog/513321) [Wasmann, 1891c](https://antcat.org/references/129643). |
| [*Formica ruficeps*](https://antcat.org/protonyms/163486) [*Formica ruficeps*](https://antcat.org/catalog/437756) | Motschoulsky, 1863 | species | unidentifiable | Unresolved junior primary homonym of [*Camponotus ruficeps*](https://antcat.org/catalog/433614) [Fabricius, 1804](https://antcat.org/references/124870) ([Bolton, 1995b](https://antcat.org/references/122860): 203). |
| [*Formica ruficornis*](https://antcat.org/protonyms/164613) [*Lasius ruficornis*](https://antcat.org/catalog/438883) | Fabricius, 1804 | species | unidentifiable | Unresolved junior primary homonym of [*Formica ruficornis*](https://antcat.org/catalog/437757) [Gmelin, 1790](https://antcat.org/references/125505). |
| [*Formica sanguinea subsp. arenicola*](https://antcat.org/protonyms/163063) [*Formica sanguinea arenicola*](https://antcat.org/catalog/437333) | Kuznetsov-Ugamsky, 1928b | subspecies | synonym | Unresolved junior primary homonym of [*Formica arenicola*](https://antcat.org/catalog/437332) [Buckley, 1866](https://antcat.org/references/123310) ([Bolton, 1995b](https://antcat.org/references/122860): 191). |
| [*Formica sanguinea subsp. monticola*](https://antcat.org/protonyms/163349) [*Formica sanguinea monticola*](https://antcat.org/catalog/437619) | Kuznetsov-Ugamsky, 1926c | subspecies | synonym | Unresolved junior primary homonym of *[Lasius monticola](https://antcat.org/catalog/438821)* [Buckley, 1866](https://antcat.org/references/123310) ([Bolton, 1995b](https://antcat.org/references/122860): 199). |
| [*Formica testacea*](https://antcat.org/protonyms/171078) [*Polyergus testaceus*](https://antcat.org/catalog/445348) | Fabricius, 1804 | species | synonym | Unresolved junior primary homonym of [*Formica testacea*](https://antcat.org/catalog/437836) [Gmelin, 1790](https://antcat.org/references/125505) ([Bolton, 1995b](https://antcat.org/references/122860): 342). |
| [*Formica vagans*](https://antcat.org/protonyms/163593) [*Formica vagans*](https://antcat.org/catalog/437863) | Fabricius, 1793 | species | unidentifiable | Unresolved junior primary homonym of *[Eciton vagans](https://antcat.org/catalog/437097)* [Olivier, 1792](https://antcat.org/references/127622) ([Bolton, 1995b](https://antcat.org/references/122860): 206). |
| [*Formica vagans*](https://antcat.org/protonyms/168989) [*Paratrechina vagans*](https://antcat.org/catalog/443259) | Jerdon, 1851 | species | synonym | Unresolved junior primary homonym of *[Eciton vagans](https://antcat.org/catalog/437097)* [Olivier, 1792](https://antcat.org/references/127622) ([Emery, 1921a](https://antcat.org/references/124747): 26; [Bolton, 1995b](https://antcat.org/references/122860): 315). |
| [*Hylidris laevigatus*](https://antcat.org/protonyms/172265) [*Pristomyrmex laevigatus*](https://antcat.org/catalog/446535) | Weber, 1952a | species | synonym | Unresolved junior secondary homonym of *[Pristomyrmex levigatus](https://antcat.org/catalog/446538)* [Emery, 1897c](https://antcat.org/references/124619). |
| [*Lasius* (*Dendrolasius*) *crispus*](https://antcat.org/protonyms/164480) [*Lasius crispus*](https://antcat.org/catalog/438750) | Wilson, 1955a | species | synonym | Unresolved junior primary homonym of [*†Lasius crispus*](https://antcat.org/catalog/438749) [Théobald, 1935a](https://antcat.org/references/129322) ([Bolton, 1995b](https://antcat.org/references/122860): 222). |
| [*Lasius emarginatus*](https://antcat.org/protonyms/202303) [*Lasius emarginatus*](https://antcat.org/catalog/513371) | Fabricius, 1804 | species | unidentifiable | Unresolved junior secondary homonym of *[Lasius emarginatus](https://antcat.org/catalog/438758)* [Olivier, 1792](https://antcat.org/references/127622). |
| [*Leptogenys* (*Lobopelta*) *nitida var. gracilis*](https://antcat.org/protonyms/164997) [*Leptogenys nitida gracilis*](https://antcat.org/catalog/439267) | Santschi, 1914e | subspecies | synonym | Unresolved junior primary homonym of [*Leptogenys gracilis*](https://antcat.org/catalog/439266) [Emery, 1899e](https://antcat.org/references/124633) ([Bolton, 1995b](https://antcat.org/references/122860): 231). |
| [*Leptothorax* (*Dichothorax*) *manni*](https://antcat.org/protonyms/175535) [*Temnothorax manni*](https://antcat.org/catalog/449805) | Wesson, 1935 | species | synonym | Unresolved junior primary homonym of [*Temnothorax manni*](https://antcat.org/catalog/449803) [Wheeler, 1914c](https://antcat.org/references/130084) ([Bolton, 1995b](https://antcat.org/references/122860): 240). |
| [*Lioponera bicolor*](https://antcat.org/protonyms/160640) [*Lioponera bicolor*](https://antcat.org/catalog/457292) | Wheeler & Chapman, 1925 | species | synonym | Unresolved junior secondary homonym of *[Lioponera bicolor](https://antcat.org/catalog/507441)* ([Bolton, 1995b](https://antcat.org/references/122860): 142). |
| [*Macromischa* (*Croesomyrmex*) *lugens var. nigra*](https://antcat.org/protonyms/175582) [*Temnothorax lugens nigra*](https://antcat.org/catalog/449852) | Santschi, 1931d | subspecies | synonym | Unresolved junior secondary homonym of [*Temnothorax niger*](https://antcat.org/catalog/449851) [Forel, 1894d](https://antcat.org/references/125061) ([Bolton, 1995b](https://antcat.org/references/122860): 241). |
| [*Macromischoides aculeatus r. inermis*](https://antcat.org/protonyms/176132) [*Tetramorium aculeatum inerme*](https://antcat.org/catalog/450402) | Bernard, 1953b | subspecies | synonym | Unresolved junior secondary homonym of [*Tetramorium inerme*](https://antcat.org/catalog/450401) [Mayr, 1877a](https://antcat.org/references/127231) ([Bolton, 1995b](https://antcat.org/references/122860): 409). |
| [*Macromischoides aculeatus var. melanogyne*](https://antcat.org/protonyms/176246) [*Tetramorium aculeatum melanogyne*](https://antcat.org/catalog/450516) | Santschi, 1923e | subspecies | synonym | Unresolved junior secondary homonym of [*Tetramorium melanogyna*](https://antcat.org/catalog/450515) [Mann, 1919](https://antcat.org/references/127071) ([Bolton, 1995b](https://antcat.org/references/122860): 411). |
| [*Macromischoides aculeatus var. pulchellus*](https://antcat.org/protonyms/176366) [*Tetramorium aculeatum pulchellum*](https://antcat.org/catalog/450636) | Santschi, 1924b | subspecies | synonym | Unresolved junior secondary homonym of [*Tetramorium pulchellum*](https://antcat.org/catalog/450634) [Emery, 1897c](https://antcat.org/references/124619) ([Bolton, 1995b](https://antcat.org/references/122860): 413). |
| [*Messor semirufus var. nigricans*](https://antcat.org/protonyms/165873) [*Messor semirufus nigricans*](https://antcat.org/catalog/440143) | Santschi, 1932c | subspecies | synonym | Unresolved junior primary homonym of *[Messor capitatus nigricans](https://antcat.org/catalog/508695)* [Santschi, 1929e](https://antcat.org/references/128384) ([Bolton, 1995b](https://antcat.org/references/122860): 255). |
| [*Myrmica* (*Monomorium*) *coeca*](https://antcat.org/protonyms/167658) [*Neivamyrmex coeca*](https://antcat.org/catalog/441928) | Buckley, 1867 | species | unidentifiable | Unresolved junior primary homonym of [*Myrmica caeca*](https://antcat.org/catalog/441400) [Jerdon, 1851](https://antcat.org/references/126168) ([Bolton, 1995b](https://antcat.org/references/122860): 288). |
| [*Myrmica* (*Monomorium*) *lineolata*](https://antcat.org/protonyms/167254) [*Myrmica lineolata*](https://antcat.org/catalog/441524) | Buckley, 1867 | species | unidentifiable | Unresolved junior primary homonym of [*Crematogaster lineolata*](https://antcat.org/catalog/435666) [Say, 1836](https://antcat.org/references/128464) ([Bolton, 1995b](https://antcat.org/references/122860): 280). |
| [*Myrmica* (*Monomorium*) *montana*](https://antcat.org/protonyms/167285) [*Myrmica montana*](https://antcat.org/catalog/441555) | Buckley, 1867 | species | unidentifiable | Unresolved junior primary homonym of [*Manica montana*](https://antcat.org/catalog/439767) [Labram & Imhoff, 1838](https://antcat.org/references/126771) ([Bolton, 1995b](https://antcat.org/references/122860): 281). |
| [*Myrmica* (*Myrmica*) *brevinodis var. transversinodis*](https://antcat.org/protonyms/176518) [*Tetramorium transversinodis*](https://antcat.org/catalog/508386) | Enzmann, 1946b | species | synonym | Unresolved junior secondary homonym of [*Tetramorium transversinode*](https://antcat.org/catalog/447281) [Mayr, 1901b](https://antcat.org/references/127247). |
| [*Myrmica* (*Myrmica*) *schencki var. subopaca*](https://antcat.org/protonyms/167402) [*Myrmica schencki subopaca*](https://antcat.org/catalog/441672) | Karavaiev, 1934 | subspecies | synonym | Unresolved junior primary homonym of [*Monomorium subopacum*](https://antcat.org/catalog/440828) [Smith, 1858a](https://antcat.org/references/128685) ([Bolton, 1995b](https://antcat.org/references/122860): 284). |
| [*Myrmica brevinodis var. subalpina*](https://antcat.org/protonyms/167398) [*Myrmica brevinodis subalpina*](https://antcat.org/catalog/441668) | Wheeler, 1917a | subspecies | synonym | Unresolved junior primary homonym of [*Myrmica kozlovi subalpina*](https://antcat.org/catalog/441667) [Ruzsky, 1915a](https://antcat.org/references/128191) ([Bolton, 1995b](https://antcat.org/references/122860): 283). |
| [*Myrmica kozlovi subsp. ruzskyi*](https://antcat.org/protonyms/167357) [*Myrmica kozlovi ruzskyi*](https://antcat.org/catalog/441627) | Weber, 1947b | subspecies | synonym | Unresolved junior primary homonym of [*Myrmica kozlovi ruzskyi*](https://antcat.org/catalog/441626) [Kiseleva, 1925](https://antcat.org/references/126440) ([Bolton, 1995b](https://antcat.org/references/122860): 282). |
| [*Myrmica laevinodis var. minuta*](https://antcat.org/protonyms/167280) [*Myrmica laevinodis minuta*](https://antcat.org/catalog/441550) | Ruzsky, 1905b | subspecies | synonym | Unresolved junior primary homonym of [*Solenopsis minuta*](https://antcat.org/catalog/447821) [Say, 1836](https://antcat.org/references/128464) ([Bolton, 1995b](https://antcat.org/references/122860): 281). |
| [*Myrmica modesta*](https://antcat.org/protonyms/176263) [*Tetramorium modesta*](https://antcat.org/catalog/450533) | Smith, 1860b | species | synonym | Unresolved junior primary homonym of [*Tetramorium modesta*](https://antcat.org/catalog/450532) [Foerster, 1850a](https://antcat.org/references/124947) ([Bolton, 1995b](https://antcat.org/references/122860): 411). |
| [*Myrmica polita*](https://antcat.org/protonyms/173611) [*Solenopsis polita*](https://antcat.org/catalog/447881) | Smith, 1862b | species | synonym | Unresolved junior primary homonym of *[Carebara polita](https://antcat.org/catalog/508344)* [Smith, 1860b](https://antcat.org/references/128691) ([Bolton, 1995b](https://antcat.org/references/122860): 390). |
| [*Myrmica rolandi var. reticulata*](https://antcat.org/protonyms/201647) [*Myrmica rolandi reticulata*](https://antcat.org/catalog/451418) | Stärcke, 1942d | subspecies | synonym | Unresolved junior primary homonym of [*Tetramorium reticulata*](https://antcat.org/catalog/450661) [Smith, 1862b](https://antcat.org/references/128696). |
| [*Myrmica rufitarsis*](https://antcat.org/protonyms/165917) [*Messor rufitarsis*](https://antcat.org/catalog/440187) | Foerster, 1850b | species | synonym | Unresolved junior secondary homonym of *[Messor rufitarsis](https://antcat.org/catalog/440186)* [Fabricius, 1804](https://antcat.org/references/124870) ([Bolton, 1995b](https://antcat.org/references/122860): 256). |
| [*Myrmica scabrinodis subsp. intermedia*](https://antcat.org/protonyms/167217) [*Myrmica scabrinodis intermedia*](https://antcat.org/catalog/441487) | Kuznetsov-Ugamsky, 1927e | subspecies | unidentifiable | Unresolved junior secondary homonym of [*†Myrmica intermedia*](https://antcat.org/catalog/441486) [Wheeler, 1915i](https://antcat.org/references/130096). |
| [*Myrmica sulcinodis var. sulcinodoscabrinodis*](https://antcat.org/protonyms/167409) [*Myrmica sulcinodis sulcinodoscabrinodis*](https://antcat.org/catalog/441679) | Forel, 1915d | subspecies | synonym | Unresolved junior primary homonym of [*Myrmica sulcinodoscabrinodis*](https://antcat.org/catalog/441678) [Ruzsky, 1895](https://antcat.org/references/128162). |
| [*Myrmica venusta*](https://antcat.org/protonyms/167436) [*Myrmica venusta*](https://antcat.org/catalog/441706) | Heer, 1867 | species | unidentifiable | Unresolved junior primary homonym of [*Monomorium venustum*](https://antcat.org/catalog/440874) [Smith, 1858a](https://antcat.org/references/128685) ([Bolton, 1995b](https://antcat.org/references/122860): 284) |
| [*Odontomachus haematodes var. fuscus*](https://antcat.org/protonyms/168227) [*Odontomachus haematodus fuscus*](https://antcat.org/catalog/442497) | Stitz, 1925c | subspecies | synonym | Unresolved junior primary homonym of [*Odontomachus assiniensis fuscus*](https://antcat.org/catalog/442496) [Stitz, 1916a](https://antcat.org/references/129026) ([Bolton, 1995b](https://antcat.org/references/122860): 295). |
| [*Oecodoma* (*Atta*) *arborea*](https://antcat.org/protonyms/160954) [*Crematogaster arborea*](https://antcat.org/catalog/435224) | Buckley, 1867 | species | synonym | Unresolved junior secondary homonym of [*Crematogaster castanea arborea*](https://antcat.org/catalog/435223) [Smith, 1858a](https://antcat.org/references/128685) ([Bolton, 1995b](https://antcat.org/references/122860): 147). |
| [*Palaeosminthurus juliae*](https://antcat.org/protonyms/158652) [*Palaeosminthurus juliae*](https://antcat.org/catalog/456815) | Pierce & Gibron, 1962 | species | unidentifiable | Note: former obsolete combination [*†Camponotus juliae*](https://antcat.org/catalog/432922) [Pierce & Gibron, 1962](https://antcat.org/references/127809) is an unresolved junior secondary homonym of [*Camponotus juliae*](https://antcat.org/catalog/432921) [Emery, 1903](https://antcat.org/references/124508). |
| [*Phasmomyrmex sericeus*](https://antcat.org/protonyms/169010) [*Camponotus sericeus*](https://antcat.org/catalog/507602) | Stitz, 1910 | species | synonym | Unresolved junior secondary homonym of [*Camponotus sericeus*](https://antcat.org/catalog/433726) [Fabricius, 1798](https://antcat.org/references/124869). |
| [*Pheidole* (*Ceratopheidole*) *hecate subsp. bruesi*](https://antcat.org/protonyms/169201) [*Pheidole hecate bruesi*](https://antcat.org/catalog/443471) | Wheeler, 1917g | subspecies | synonym | Unresolved junior primary homonym of [*Pheidole bruesi*](https://antcat.org/catalog/443470) [Wheeler, 1911b](https://antcat.org/references/130056) ([Bolton, 1995b](https://antcat.org/references/122860): 318). |
| [*Pheidole cubaensis var. grayi*](https://antcat.org/protonyms/169561) [*Pheidole cubaensis grayi*](https://antcat.org/catalog/443831) | Mann, 1920b | subspecies | synonym | Unresolved junior primary homonym of [*Pheidole grayi*](https://antcat.org/catalog/443830) [Forel, 1902c](https://antcat.org/references/125121) ([Bolton, 1995b](https://antcat.org/references/122860): 322). |
| [*Pheidole inquilina*](https://antcat.org/protonyms/169665) [*Pheidole inquilina*](https://antcat.org/catalog/443935) | Forel, 1914d | species | synonym | Unresolved junior secondary homonym of [*Pheidole inquilina*](https://antcat.org/catalog/443934) [Wheeler, 1903h](https://antcat.org/references/129981) ([Bolton, 1995b](https://antcat.org/references/122860): 323). |
| [*Pheidole laevigata*](https://antcat.org/protonyms/169718) [*Pheidole laevigata*](https://antcat.org/catalog/443988) | Mayr, 1862 | species | synonym | Unresolved junior secondary homonym of [*Pheidole laevigata*](https://antcat.org/catalog/443987) [Smith, 1855b](https://antcat.org/references/128680). |
| [*Pheidole rhombinoda var. taprobanae*](https://antcat.org/protonyms/170364) [*Pheidole rhombinoda taprobanae*](https://antcat.org/catalog/444634) | Forel, 1902c | subspecies | synonym | Unresolved junior primary homonym of *[Carebara diversa taprobanae](https://antcat.org/catalog/462036)* [Smith, 1858a](https://antcat.org/references/128685) ([Bolton, 1995b](https://antcat.org/references/122860): 331). |
| [*Pheidologeton australis var. mjobergi*](https://antcat.org/protonyms/170561) [*Carebara australis mjobergi*](https://antcat.org/catalog/508666) | Forel, 1918a | subspecies | synonym | Unresolved junior secondary homonym of *[Carebara mjobergi](https://antcat.org/catalog/434272)* [Forel, 1915b](https://antcat.org/references/125258). |
| [*Phyracaces hewitti*](https://antcat.org/protonyms/160703) [*Lioponera hewitti*](https://antcat.org/catalog/508190) | Donisthorpe, 1931d | species | synonym | Unresolved junior primary homonym of *[Lioponera hewitti](https://antcat.org/catalog/507479)* [Wheeler, 1919f](https://antcat.org/references/130136) ([Bolton, 1995b](https://antcat.org/references/122860): 143). |
| [*Polyrhachis* (*Cyrtomyrma*) *rastellata var. ceylonensis*](https://antcat.org/protonyms/171253) [*Polyrhachis rastellata ceylonensis*](https://antcat.org/catalog/445523) | Donisthorpe, 1938c | subspecies | synonym | Unresolved junior primary homonym of [*Polyrhachis hippomanes ceylonensis*](https://antcat.org/catalog/445522) [Emery, 1893h](https://antcat.org/references/124568) ([Bolton, 1995b](https://antcat.org/references/122860): 345). |
| [*Polyrhachis bihamata var. minor*](https://antcat.org/protonyms/171670) [*Polyrhachis bihamata minor*](https://antcat.org/catalog/445940) | Karavaiev, 1927f | subspecies | synonym | Unresolved junior primary homonym of [*Polyrhachis armata minor*](https://antcat.org/catalog/445939) [Forel, 1886d](https://antcat.org/references/125013) ([Bolton, 1995b](https://antcat.org/references/122860): 352). |
| [*Polyrhachis conops var. cuspidata*](https://antcat.org/protonyms/171327) [*Polyrhachis conops cuspidata*](https://antcat.org/catalog/445597) | Stitz, 1911a | subspecies | synonym | Unresolved junior primary homonym of [*Dolichoderus cuspidatus*](https://antcat.org/catalog/436525) [Smith, 1857a](https://antcat.org/references/128683) ([Bolton, 1995b](https://antcat.org/references/122860): 346). |
| [*Ponera* (*Hypoponera*) *intermedia*](https://antcat.org/protonyms/164073) [*Hypoponera intermedia*](https://antcat.org/catalog/438343) | Bernard, 1953b | species | synonym | Unresolved junior primary homonym of *[Pseudoneoponera piliventris intermedia](https://antcat.org/catalog/442938)* [Forel, 1900b](https://antcat.org/references/125097) ([Bolton, 1995b](https://antcat.org/references/122860): 215). |
| [*Ponera* (*Hypoponera*) *jeannebi st. abyssinica*](https://antcat.org/protonyms/163972) [*Hypoponera abyssinica*](https://antcat.org/catalog/511274) | Santschi, 1938b | species | synonym | Unresolved junior primary homonym of *[Megaponera abyssinica](https://antcat.org/catalog/442751)* [Guérin-Méneville, 1849](https://antcat.org/references/125707) ([Bolton, 1995b](https://antcat.org/references/122860): 213). |
| [*Ponera affinis*](https://antcat.org/protonyms/172071) [*Ponera affinis*](https://antcat.org/catalog/446341) | Jerdon, 1851 | species | unidentifiable | Unresolved junior primary homonym of [*†Liometopum affine*](https://antcat.org/catalog/508888) [Heer, 1849](https://antcat.org/references/125815) ([Bolton, 1995b](https://antcat.org/references/122860): 360). |
| [*Ponera bicolor*](https://antcat.org/protonyms/168528) [*Brachyponera bicolor*](https://antcat.org/catalog/442798) | Donisthorpe, 1949b | species | synonym | Unresolved junior primary homonym of *[Neoponera bicolor](https://antcat.org/catalog/442797)* [Guérin-Méneville, 1844a](https://antcat.org/references/125711) ([Bolton, 1995b](https://antcat.org/references/122860): 303). |
| [*Ponera caeca*](https://antcat.org/protonyms/172353) [*Proceratium caeca*](https://antcat.org/catalog/446623) | Donisthorpe, 1949b | species | synonym | Unresolved junior primary homonym of *[Hypoponera coeca](https://antcat.org/catalog/438277)* [Santschi, 1914d](https://antcat.org/references/128288) ([Bolton, 1995b](https://antcat.org/references/122860): 366). |
| [*Ponera ergatandria st. cognata*](https://antcat.org/protonyms/164008) [*Hypoponera cognata*](https://antcat.org/catalog/509334) | Santschi, 1912b | species | synonym | Unresolved junior primary homonym of *[Pseudoponera cognata](https://antcat.org/catalog/442833)* [Emery, 1896g](https://antcat.org/references/124611) ([Bolton, 1995b](https://antcat.org/references/122860): 213). |
| [*Ponera gleadowi subsp. aethiopica*](https://antcat.org/protonyms/163974) [*Hypoponera aethiopica*](https://antcat.org/catalog/511275) | Forel, 1907f | species | synonym | Unresolved junior primary homonym of *[Streblognathus aethiopicus](https://antcat.org/catalog/448229)* [Smith, 1858a](https://antcat.org/references/128685) ([Bolton, 1995b](https://antcat.org/references/122860): 213). |
| [*Ponera japonica var. crocea*](https://antcat.org/protonyms/172090) [*Ponera japonica crocea*](https://antcat.org/catalog/446360) | Santschi, 1941 | subspecies | synonym | Unresolved junior primary homonym of *[Proceratium croceum](https://antcat.org/catalog/446638)* [Roger, 1860](https://antcat.org/references/128087) ([Bolton, 1995b](https://antcat.org/references/122860): 360). |
| [*Prenolepis steinheili var. minuta*](https://antcat.org/protonyms/168024) [*Nylanderia steinheili minuta*](https://antcat.org/catalog/442294) | Forel, 1893j | subspecies | synonym | Unresolved junior primary homonym of *[Prenolepis imparis minuta](https://antcat.org/catalog/446466)* [Emery, 1893k](https://antcat.org/references/124571) ([Bolton, 1995b](https://antcat.org/references/122860): 314). |
| [*Pseudomyrma belti subsp. bequaerti*](https://antcat.org/protonyms/172690) [*Pseudomyrmex belti bequaerti*](https://antcat.org/catalog/446960) | Enzmann, 1944 | subspecies | synonym | Unresolved junior primary homonym of *[Pseudomyrmex belti bequaerti](https://antcat.org/catalog/446959)* [Wheeler, 1942](https://antcat.org/references/129956). |
| [*Pseudomyrma bradleyi*](https://antcat.org/protonyms/172700) [*Pseudomyrmex bradleyi*](https://antcat.org/catalog/446970) | Enzmann, 1944 | species | synonym | Unresolved junior primary homonym of *[Pseudomyrmex latinodus bradleyi](https://antcat.org/catalog/446969)* [Wheeler, 1942](https://antcat.org/references/129956). |
| [*Pseudomyrma gracilis var. peruviana*](https://antcat.org/protonyms/172871) [*Pseudomyrmex gracilis peruviana*](https://antcat.org/catalog/447141) | Enzmann, 1944 | subspecies | synonym | Unresolved junior primary homonym of *[Pseudomyrmex peruvianus](https://antcat.org/catalog/447140)* [Wheeler, 1925a](https://antcat.org/references/130175) ([Bolton, 1995b](https://antcat.org/references/122860): 375). |
| [*Pseudomyrma latinoda var. coronata*](https://antcat.org/protonyms/172725) [*Pseudomyrmex latinodus coronatus*](https://antcat.org/catalog/446995) | Enzmann, 1944 | subspecies | synonym | Unresolved junior primary homonym of *[Pseudomyrmex coronatus](https://antcat.org/catalog/446994)* [Wheeler, 1942](https://antcat.org/references/129956). |
| [*Pseudomyrma longiceps*](https://antcat.org/protonyms/172825) [*Pseudomyrmex longiceps*](https://antcat.org/catalog/447095) | Stitz, 1933 | species | synonym | Unresolved junior primary homonym of *[Pseudomyrmex biconvexa longiceps](https://antcat.org/catalog/447094)* [Forel, 1906d](https://antcat.org/references/125167) ([Bolton, 1995b](https://antcat.org/references/122860): 374). |
| [*Pseudomyrma sabanica var. saffordi*](https://antcat.org/protonyms/172889) [*Pseudomyrmex satanicus saffordi*](https://antcat.org/catalog/447159) | Enzmann, 1944 | subspecies | synonym | Unresolved junior primary homonym of *[Pseudomyrmex belti saffordi](https://antcat.org/catalog/447158)* [Wheeler, 1942](https://antcat.org/references/129956) ([Bolton, 1995b](https://antcat.org/references/122860): 375). |
| [*Pseudomyrma spinolae var. infernalis*](https://antcat.org/protonyms/172798) [*Pseudomyrmex spinicola infernalis*](https://antcat.org/catalog/509632) | Enzmann, 1944 | subspecies | synonym | Unresolved junior primary homonym of *[Pseudomyrmex spinicola infernalis](https://antcat.org/catalog/447067)* [Wheeler, 1942](https://antcat.org/references/129956). |
| [*Pseudomyrma spinolae var. scelerosa*](https://antcat.org/protonyms/172895) [*Pseudomyrmex spinicola scelerosus*](https://antcat.org/catalog/509633) | Enzmann, 1944 | subspecies | synonym | Unresolved junior primary homonym of *[Pseudomyrmex spinicola scelerosus](https://antcat.org/catalog/447164)* [Wheeler, 1942](https://antcat.org/references/129956). |
| [*Pseudomyrma triplaridis subsp. boxi*](https://antcat.org/protonyms/172698) [*Pseudomyrmex triplaridis boxi*](https://antcat.org/catalog/446968) | Enzmann, 1944 | subspecies | synonym | Unresolved junior primary homonym of *[Pseudomyrmex triplaridis boxi](https://antcat.org/catalog/446967)* [Wheeler, 1942](https://antcat.org/references/129956). |
| [*Rhytidoponera petiolata*](https://antcat.org/protonyms/173124) [*Rhytidoponera petiolata*](https://antcat.org/catalog/447394) | Clark, 1936 | species | synonym | Unresolved junior primary homonym of *[Rhytidoponera laciniosa petiolata](https://antcat.org/catalog/447393)* [Viehmeyer, 1912b](https://antcat.org/references/129525) ([Bolton, 1995b](https://antcat.org/references/122860): 380). |
| [*Santschia intrudens*](https://antcat.org/protonyms/201156) [*Monomorium intrudens*](https://antcat.org/catalog/509095) | Forel, 1906a | species | synonym | Unresolved junior secondary homonym of [*Monomorium intrudens*](https://antcat.org/catalog/440564) [Smith, 1874b](https://antcat.org/references/128712). |
| [*Tapinoma boreale*](https://antcat.org/protonyms/174999) [*Tapinoma boreale*](https://antcat.org/catalog/449269) | Provancher, 1887b | species | synonym | Unresolved junior primary homonym of [*Tapinoma boreale*](https://antcat.org/catalog/449268) [Roger, 1863a](https://antcat.org/references/128093): [Dalla Torre, 1893](https://antcat.org/references/124002): 164. |
| [*Technomyrmex albipes subsp. congolensis*](https://antcat.org/protonyms/175138) [*Technomyrmex albipes congolensis*](https://antcat.org/catalog/449408) | Karavaiev, 1926d | subspecies | synonym | Unresolved junior secondary homonym of *[Technomyrmex laurenti congolensis](https://antcat.org/catalog/449407)* [Forel, 1916](https://antcat.org/references/124996) ([Bolton, 1995b](https://antcat.org/references/122860): 402). |
| [*Technomyrmex modiglianii r. javanus*](https://antcat.org/protonyms/175174) [*Technomyrmex modiglianii javanus*](https://antcat.org/catalog/449444) | Forel, 1905f | subspecies | synonym | Unresolved junior secondary homonym of *[Technomyrmex setiferum javanum](https://antcat.org/catalog/449443)* [Forel, 1905f](https://antcat.org/references/125163) ([Bolton, 1995b](https://antcat.org/references/122860): 402). |
| [*Tetramorium* (*Triglyphothrix*) *auropunctatus var. bulawayensis*](https://antcat.org/protonyms/175925) [*Tetramorium auropunctatus bulawayense*](https://antcat.org/catalog/450195) | Arnold, 1917 | subspecies | synonym | Unresolved junior primary homonym of [*Tetramorium bulawayense*](https://antcat.org/catalog/450194) [Forel, 1913a](https://antcat.org/references/125238) ([Bolton, 1995b](https://antcat.org/references/122860): 405). |
| [*Tetramorium* (*Triglyphothrix*) *marleyi var. akermani*](https://antcat.org/protonyms/175832) [*Tetramorium marleyi akermani*](https://antcat.org/catalog/450102) | Arnold, 1926 | subspecies | synonym | Unresolved junior primary homonym of [*Tetramorium akermani*](https://antcat.org/catalog/450101) [Arnold, 1926](https://antcat.org/references/122314) ([Bolton, 1995b](https://antcat.org/references/122860): 404). |
| [*Tetramorium do var. mus*](https://antcat.org/protonyms/176272) [*Tetramorium do mus*](https://antcat.org/catalog/450542) | Arnold, 1960a | subspecies | synonym | Unresolved junior secondary homonym of [*Tetramorium guillodi mus*](https://antcat.org/catalog/450541) [Santschi, 1937d](https://antcat.org/references/128434) ([Bolton, 1995b](https://antcat.org/references/122860): 411). |
| [*Tetramorium grazsii var. mayri*](https://antcat.org/protonyms/176241) [*Tetramorium grassii mayri*](https://antcat.org/catalog/450511) | Emery, 1924f | subspecies | synonym | Unresolved junior secondary homonym of [*Tetramorium mayri*](https://antcat.org/catalog/450510) [Forel, 1912o](https://antcat.org/references/125237) ([Bolton, 1995b](https://antcat.org/references/122860): 411). |
| [*Tetramorium semireticulatum var. politum*](https://antcat.org/protonyms/176356) [*Tetramorium semireticulatum politum*](https://antcat.org/catalog/450626) | Arnold, 1948 | subspecies | synonym | Unresolved junior primary homonym of [*Tetramorium politum*](https://antcat.org/catalog/450625) [Emery, 1897](https://antcat.org/references/133003) ([Bolton, 1995b](https://antcat.org/references/122860): 413). |
| [*Tetramorium simillimum var. insulare*](https://antcat.org/protonyms/176141) [*Tetramorium simillimum insulare*](https://antcat.org/catalog/450411) | Santschi, 1928c | subspecies | synonym | Unresolved junior secondary homonym of [*Tetramorium insulare*](https://antcat.org/catalog/450410) [Menozzi, 1924b](https://antcat.org/references/127321) ([Bolton, 1995b](https://antcat.org/references/122860): 409). |
| [*Triglyphothrix striatidens var. flavescens*](https://antcat.org/protonyms/176051) [*Tetramorium striatidens flavescens*](https://antcat.org/catalog/450321) | Wheeler, 1929h | subspecies | synonym | Unresolved junior secondary homonym of [*Tetramorium costatus flavescens*](https://antcat.org/catalog/450320) [Emery, 1897c](https://antcat.org/references/124619) ([Bolton, 1995b](https://antcat.org/references/122860): 407). |
| [*Wasmannia auropunctata subsp. brevispinosa*](https://antcat.org/protonyms/197604) [*Tetramorium auropunctatus brevispinosum*](https://antcat.org/catalog/451485) | Borgmeier, 1928a | subspecies | synonym | Unresolved junior secondary homonym of [*Tetramorium brevispinosum*](https://antcat.org/catalog/450189) [Stitz, 1910](https://antcat.org/references/129021) ([Bolton, 1995b](https://antcat.org/references/122860): 405). |
| [*Xenoaphaenogaster inquilina*](https://antcat.org/protonyms/169666) [*Pheidole inquilina*](https://antcat.org/catalog/443936) | Baroni Urbani, 1964b | species | synonym | Unresolved junior secondary homonym of [*Pheidole inquilina*](https://antcat.org/catalog/443934) [Wheeler, 1903h](https://antcat.org/references/129981) ([Bolton, 1995b](https://antcat.org/references/122860): 323). |
| [*Xiphomyrmex tricolor*](https://antcat.org/protonyms/176521) [*Tetramorium tricolor*](https://antcat.org/catalog/450791) | Donisthorpe, 1949c | species | synonym | Unresolved junior secondary homonym of [*Tetramorium tricolor*](https://antcat.org/catalog/450790) [Donisthorpe, 1948g](https://antcat.org/references/124363) ([Bolton, 1995b](https://antcat.org/references/122860): 415). |
| [*Xiphomyrmex uelensis*](https://antcat.org/protonyms/176536) [*Tetramorium uelense*](https://antcat.org/catalog/450806) | Santschi, 1935a | species | synonym | Unresolved junior primary homonym of [*Tetramorium uelense*](https://antcat.org/catalog/457449) [Santschi, 1923e](https://antcat.org/references/128347) ([Bolton, 1995b](https://antcat.org/references/122860): 416). |
